# Integrated Single-Cell and Spatial Transcriptomic Analysis Identifies Putative Metabolic Crosstalk Between *SPP1*+ TAMs and *SLC6A20*+ Epithelial Cells in Colorectal Cancer

**DOI:** 10.3390/cancers18111755

**Published:** 2026-05-27

**Authors:** Yu Xue, Guangsong Tang, Xinglong Li, Qingfa Wu, Weiqiang Yu

**Affiliations:** 1 School of Life Sciences, Faculty of Medicine, Tianjin University, Tianjin 300072, China; xueyuu@tju.edu.cn; 2 HIM-BGI Omics Center, Zhejiang Cancer Hospital, Hangzhou Institute of Medicine (HIM), Chinese Academy of Sciences (CAS), Hangzhou 310022, China; tgs@zjut.edu.cn (G.T.); lixl@zjut.edu.cn (X.L.); 3 College of Computer Science & Technology, Zhejiang University of Technology, Hangzhou 310023, China; 4 College of Pharmaceutical Science, Zhejiang University of Technology, Hangzhou 310014, China; 5 Center for Advanced Interdisciplinary Science and Biomedicine of IHM, Division of Life Sciences and Medicine, University of Science and Technology of China, Hefei 230027, China; 6 Department of Pharmacy, The First Affiliated Hospital of USTC, Division of Life Sciences and Medicine, University of Science and Technology of China, Hefei 230001, China

**Keywords:** colorectal cancer, scRNA-seq, spatial transcriptomics, metabolic reprogramming, metabolic crosstalk, prognostic model

## Abstract

**Background**: Colorectal cancer (CRC) progression is associated with tumor metabolic reprogramming and an immunosuppressive tumor microenvironment, yet coordinated metabolic interactions between malignant epithelial and immune cells remain unclear. This study aimed to characterize metabolic crosstalk in CRC, validate spatial organization, and develop a metabolism-based prognostic model. **Methods**: Six CRC single-cell RNA sequencing datasets were integrated to identify cell populations, evaluate metabolic pathway activity, and infer cell–cell communication. Spatial transcriptomics was used to assess regional co-enrichment of key cell-subset signatures and metabolic activities. Bulk transcriptomic cohorts and targeted metabolomics data were analyzed for pathway-level support. Patients were stratified using metabolic features of selected subsets, followed by protein–protein interaction analysis and elastic net modeling. **Results**: Across six scRNA-seq datasets comprising 431,217 cells from 173 samples (107 tumor, 60 normal, and 6 border), we identified a metabolically reprogrammed malignant epithelial subset (*SLC6A20*+ epithelial cells) and an immunosuppressive *SPP1*+ tumor-associated macrophage (TAM) subset. Both exhibited elevated glycolysis, vitamin B6 metabolism, and aromatic amino acid metabolism. Spatial transcriptomics supported regional co-enrichment of their signatures and shared metabolic activities within the same tumor regions. Independent bulk transcriptomic cohorts and targeted metabolomics further supported these pathway alterations. Cell–cell communication analysis revealed extensive bidirectional ligand-receptor interactions. Based on metabolic features of these subsets, patients were stratified into two prognostic groups. A 14-gene elastic net signature predicted the high-risk subtype with consistent performance across independent cohorts. **Conclusions**: *SLC6A20*+ epithelial cells and *SPP1*+ TAMs showed coordinated, transcriptome-inferred metabolic programs and predicted bidirectional communication in CRC. These features provide candidate biologically interpretable biomarkers and a metabolism-based prognostic model for patient stratification.

## Simple Summary

Colorectal cancer cells do not grow alone; they interact with nearby immune cells and can change how nutrients are used inside tumors. These interactions may help tumors grow, avoid immune attack, and lead to worse outcomes, but they are not fully understood. In this study, we examined how cancer epithelial cells and tumor-associated macrophages communicate through metabolic programs using single-cell and spatial analyses. We identified a closely linked pair of cell populations, *SLC6A20*+ epithelial cells and *SPP1*+ TAMs, that shared altered energy, vitamin B6, and amino acid metabolism. Their signals were found in the same tumor regions and were associated with poorer prognosis. Based on these findings, we developed a gene-based model that may help classify patients into different risk groups and support future research on metabolism-related treatment strategies.

## 1. Introduction

Colorectal cancer (CRC) is the third most prevalent cancer worldwide and the second leading cause of cancer-related mortality, with high lethality and recurrence rates; by 2035, CRC is expected to result in 2.5 million new cases, underscoring the urgency of addressing this disease [1,2]. Although CRC screening with fecal immunochemical testing and colonoscopy is essential for prevention, suboptimal uptake and recent evidence on screening knowledge and participation among healthcare staff highlight ongoing implementation gaps; alongside these efforts, a deeper understanding of CRC progression remains necessary for prognostic assessment and therapeutic decision-making [3,4]. Tumor development involves complex genetic, environmental, and metabolic interactions, with metabolic reprogramming as a critical hallmark [5]. To sustain proliferation, tumor cells remodel metabolism to meet energy demands, support macromolecule biosynthesis, and counteract oxidative stress in the tumor microenvironment (TME) [6]. CRC is also characterized by marked intratumoral heterogeneity, where distinct cellular subpopulations exhibit divergent metabolic programs that influence therapeutic responses and metastatic potential [7].

Advances in single-cell transcriptomics have revolutionized our understanding of CRC heterogeneity and the complexity of the TME [8]. These technologies enable high-resolution dissection of metabolic states, signaling pathways, and intercellular interactions across tumor and stromal compartments [9]. Despite this progress, the specific mechanisms by which cell–cell crosstalk drives metabolic dysregulation remain incompletely understood, highlighting an urgent need for single-cell studies that resolve cell-specific metabolic dependencies [10]. However, many tumor-immune interactions are spatially organized, making spatial transcriptomics a valuable in situ validation of single-cell findings.

Within the TME, tumor-associated macrophages (TAMs) are pivotal regulators of CRC progression and serve as important prognostic indicators [11]. The M2-polarized TAM phenotype, predominant in most tumors, facilitates immune evasion and promotes malignancy [12]. Strikingly, M2 macrophages and tumor cells often share hyperactive metabolic pathways and key metabolites, suggesting a metabolic convergence that underpins their cooperative roles in tumor progression [13].

Here, we integrate multiple publicly available single-cell transcriptomic datasets of CRC to systematically delineate the cellular landscape and metabolic architecture of the TME. We identify a tumor-promoting macrophage subset (*SPP1*+ TAMs) and a malignant epithelial subset (*SLC6A20*+ epithelial cells) that exhibit coordinated metabolic coupling and inferred ligand-receptor signaling, and are associated with pro-tumoral and immunosuppressive phenotypes. Both subsets exhibit upregulated glycolysis, vitamin B6 metabolism, and aromatic amino acid biosynthesis pathways associated with immune evasion mediated by lactate accumulation and immunosuppressive metabolites such as kynurenine [14,15]. We further used spatial transcriptomics for in situ mapping, supporting regional co-enrichment of *SLC6A20*+ epithelial cell and *SPP1*+ TAM signatures and enrichment of key metabolic programs within these regions. These findings suggest the *SPP1*+ TAMs-*SLC6A20*+ epithelial cells metabolic axis as a potential therapeutic vulnerability in CRC. Moreover, we developed a prognostic model that utilizes the metabolic interactions between *SPP1*+ TAMs and *SLC6A20*+ epithelial cells to stratify CRC patients into distinct subtypes. We also validated the model in the other three validation cohorts. The model showed robust predictive performance, providing a promising tool for personalized prognosis and decision-making in CRC.

## 2. Materials and Methods

### 2.1. Data Source

We obtained six single-cell RNA sequencing (scRNA-seq) datasets (GSE178341, GSE132257, GSE132465, GSE144735, GSE188711, and GSE231559) from the Gene Expression Omnibus (GEO; https://www.ncbi.nlm.nih.gov/geo/; accessed on 12 May 2026). These datasets included 107 colorectal cancer (CRC) samples, 60 normal tissue samples, and 6 border tissue samples, enabling comparative analyses between tumor and non-tumor tissues. Transcriptional profiles and corresponding clinical data for the TCGA-COAD cohort were retrieved from the UCSC Xena platform (http://xena.ucsc.edu/; accessed on 12 May 2026) and used for survival analysis and prognostic model development. The prognostic model was further validated in three independent cohorts (GSE38832, GSE39582, and GSE17538). In addition, two bulk CRC datasets (GSE44076 and GSE113513) were used to validate key metabolic genes, spatial transcriptomics data from GSE225857 were used for in situ validation, and targeted metabolomics data from the MetaboLights (https://www.ebi.ac.uk/metabolights/index; accessed on 12 May 2026) dataset MTBLS8090 were used for metabolite-level validation. The accession numbers, dataset URLs, sample composition, and use of all public datasets are summarized in Supplementary Table S1.

### 2.2. Data Merging, Quality Control, and Normalization

Using the GRCh38 reference genome (https://www.10xgenomics.com/support; accessed on 12 May 2026), we identified shared genes across all six datasets, which were merged using Scanpy (version 1.10.3) with the anndata.concat function from anndata (version 0.11.1; join = ‘inner’) to generate a joint expression matrix. Cells with <200 or >5000 detected genes, total UMI counts <200 or >50,000, mitochondrial gene expression > 30%, ribosomal gene expression < 5%, or hemoglobin gene expression > 1% were filtered out, leaving 431,217 cells. Raw counts were depth-normalized to 10,000 total counts and transformed with the ‘log1p’ function in scanpy.

### 2.3. Integration, Dimensionality Reduction, and Clustering

Given the large size of the dataset and its multi-sample design, we integrated samples using scANVI to mitigate sample-associated technical variation while preserving biological differences and minimizing over-correction. We first identified the top 2000 highly variable genes (HVGs) using sc.pp.highly_variable_genes. These HVGs were used as input to scVI (version 1.2.1) (with batch_key = ‘Sample’) to correct for batch effects. Low-dimensional representations learned by scVI were subsequently processed in Scanpy to construct a neighborhood graph, perform Uniform Manifold Approximation and Projection (UMAP) dimensionality reduction, and apply the Leiden algorithm across a range of resolutions for clustering. At each resolution, differential expression analysis was performed using sc.tl.rank_genes_groups function to identify cluster-specific marker genes, and cell types were manually annotated based on canonical markers. Then, the best resolution was identified according to differential expression genes in each cluster.

To refine annotations, scANVI was initialized from the pretrained scVI model, trained for up to 20 epochs with 100 cells sampled per label, and its latent embeddings (adata.obsm[‘X_scANVI’]) were used for UMAP visualization. Clustering was performed in two stages: in the first stage, cells were assigned to major celltypes, including T cells (*CD3D*, *CD3E*, *CD8A*; n = 128,118), B cells (*MS4A1*, *CD19*; n = 39,971), plasma cells (*CD79A*, *JCHAIN*, *MZB1*; n = 46,803), mast cells (*TPSAB1*, *GATA2*; n = 5277), myeloid cells (*CD14*, *FCGR3A*, *C1QA*; n = 52,783), endothelial cells (*CLDN5*, *VWF*; n = 10,545), fibroblasts (*THY1*, *DCN*, *ACTA2*; n = 20,010), glial cells (*PLP1*, *SOX10*, *PTPRZ1*; n = 1140), and epithelial cells (*EPCAM*, *KRT18*, *KRT19*; n = 126,570). In the second stage, myeloid and epithelial compartments were further subdivided into transcriptionally distinct subtypes. Then, we also annotated each cluster as in the previous step to ensure accurate classification.

### 2.4. Copy Number Variation Analysis

The infercnv (version 1.18.1) package was used to infer large-scale copy number variations (CNVs) from single-cell gene expression data. As normal copy-number controls, 500 T cells, 500 myeloid cells, and 500 fibroblasts were randomly selected. By evaluating iterative clustering results and computing CNV scores, normal epithelial subpopulations were distinguished from tumor epithelial subpopulations.

### 2.5. Pathway Enrichment Analysis

Human KEGG pathway representation analysis was performed using the enrichKEGG function from the clusterProfiler (version 4.6.2) package on the differentially expressed genes (DEGs) of Macro_SPP1, Mono_EREG, and Mono_HSPA1A, as well as on the downstream targets of *SPP1*+ TAMs to *SLC6A20*+ epithelial cells predicted by NicheNet.

For the DEGs of *SPP1*+ macrophages, Mono_EREG, and Mono_HSPA1A, Gene Set Enrichment Analysis (GSEA) was conducted using the GSEA function from the clusterProfiler package with 82 KEGG metabolic pathway gene sets, which were downloaded from the KEGG PATHWAY Database (https://www.kegg.jp/kegg/pathway.html; accessed on 12 May 2026).

Additionally, Gene Ontology (GO) enrichment analysis was performed on the NicheNet-predicted downstream targets of *SPP1*+ TAMs to *SLC6A20*+ epithelial cells using the enrichGO function from clusterProfiler. The GO.db (version 3.16.0) package was then used to extract metabolic process pathways (GO:0008152) and their associated offspring terms.

### 2.6. Gene Signature Scoring

Hallmark gene sets were downloaded from the Molecular Signatures Database (MSigDB; http://software.broadinstitute.org/gsea/msigdb/; accessed on 12 May 2026). Macrophage polarization-associated gene signatures were from Cheng et al. [16]. Subsequently, we applied the AddModuleScore function from Seurat (version 4.4.0) to calculate module scores for all the aforementioned gene signatures. Briefly, for each cell, the score is the mean of the scaled expression values of all genes in the signature.

### 2.7. Trajectory Analysis

First, the CytoTRACE (version 0.3.3) algorithm was applied to assess the stemness potential of myeloid cell subsets. CytoTRACE predicts cellular differentiation potential by calculating stemness scores and determines each cell’s position along the differentiation trajectory. Next, we employed the Partition-based Graph Abstraction (PAGA) method to construct a topological graph of myeloid cell subsets, quantify inter-subcluster connectivity, and infer cell state transition paths based on node and edge weights to identify potential differentiation branches. By integrating CytoTRACE and PAGA results, we mapped the developmental trajectory of myeloid cells.

### 2.8. CRC Specific Signature Gene Analysis

CRC tumor and matched adjacent normal tissue transcriptomic data from The Cancer Genome Atlas (TCGA) database were used for validation of the CNV analysis results. Differential expression analysis was conducted to identify differentially expressed genes (DEGs) between tumor and normal samples. Two gene sets were subsequently defined. The TumorgeneSet comprises 30 genes that are significantly upregulated in tumor samples. The NormalgeneSet contains 30 genes that are significantly upregulated in adjacent normal tissues (Supplementary Table S2).

### 2.9. Transcription Factor Regulon Analysis

SCENIC analysis was performed with pyscenic (version 0.12.1) to infer transcription factor (TF) regulons and quantify their activity in myeloid cells. Raw counts from myeloid subsets were normalized to 10,000 counts per cell, log-transformed, and the top 5000 highly variable genes were exported as a LOOM file with gene and cell annotations.

Regulatory network inference was conducted using the GRNBoost2 algorithm with a curated list of human TFs (hs_hgnc_tfs.txt). For motif enrichment analysis, the co-expression modules were evaluated against the motif database, and enriched motifs were annotated using motif-v9-nr.hgnc (m0.001-o0.0). Regulon activity scores were calculated per cell using the AUCell algorithm.

The resulting regulon activity matrix was imported into R (version 4.2.2) via SCopeLoomR (version 0.13.0) for visualization. Regulon specificity scores (RSS) were computed to assess cell type-specific TF activity, and heatmaps or Violin plots were generated to display AUCell scores across myeloid cell types.

### 2.10. scMetabolism-Based Metabolic Pathway Activity Analysis

Metabolic pathway activity analysis was performed using the scMetabolism (version 0.2.1) package with 82 KEGG metabolic pathways as input on single-cell data from myeloid and epithelial cell subsets. The data were first normalized, followed by the selection of HVGs, log transformation, and Z-score scaling. Principal component analysis (PCA) was then conducted. Subsequently, the sc.metabolism.Seurat function was employed to calculate the metabolic pathway activity scores for each cell based on 82 KEGG metabolic pathways, utilizing the AUCell method. No imputation was performed (imputation = F), and the calculations were parallelized using 10 cores. The computed metabolic pathway activity scores were merged into the metadata of the Seurat object. Importantly, scMetabolism scores represent gene expression-derived estimates of pathway activity and do not directly measure metabolite abundance, enzymatic activity, or metabolic flux. Accordingly, these scores were interpreted as transcriptionally inferred metabolic programs rather than direct evidence of metabolite-level changes or metabolic flux.

To further explore metabolic activity, we selected the top half of the pathways with the highest overall activity for additional analysis. The relative metabolic activity of each cell subtype was calculated, and the DotPlot function was used to illustrate the relationship between metabolic pathway activity and subcell types, highlighting differences in metabolic activity across subpopulations. Finally, a boxplot was generated using ggplot2 (version 3.4.4) to visualize the distribution of metabolic pathway activity across cell subtypes. Cell subtypes were ranked according to metabolic activity, further elucidating the distinct metabolic characteristics of each subtype.

### 2.11. Intercellular Interactions and Ligand–Receptor Analysis

CellPhoneDB (version 5.0.1) is a publicly available database of curated ligand–receptor interactions and can be used to query specific ligand/receptor pairs. For differential analysis of tumor-normal cell communication (colorectal cancer vs. normal tissue), we separately constructed interaction networks between *SPP1*+ TAMs and Epi01 (*SLC6A20*+ epithelial cells) in colorectal cancer and in adjacent normal samples, and visualized the significantly differential ligand–receptor pairs using the R package circlize (version 0.4.16).

NicheNet can predict signaling networks between different cell populations by integrating scRNA-seq data with a curated ligand–receptor target pathway database to infer potential cell–cell communication. We used nichenetr (version 2.2.0) package to infer the interactions between the *SLC6A20*+ epithelial cells and *SPP1*+ TAMs. Using Nichen-et_output$ligand_activity_target_heatmap, we plotted a heatmap of ligand regulatory activity scores ranging from 0 to 1.5; differential expression of ligands and receptors was also visualized as heatmaps by computing average gene expression per cell type and normalizing across the indicated subtypes.

### 2.12. ST Analysis, Tangram Mapping, Metabolic and Ligand–Receptor Characterization In Situ

Spatial transcriptomics data from three CRC sections (C2, C3, and C4; GSE225857) were analyzed in Scanpy. Given that 10× Genomics Visium spots are approximately 55 μm in diameter and can capture transcripts from multiple cells, spatial patterns were interpreted as regional co-enrichment rather than direct physical interactions at single-cell resolution. Spots with fewer than 200 detected genes were excluded. The data were normalized to 10,000 counts per spot, log-transformed, and highly variable genes were selected (top 2000). Sections were batch-corrected using Scanorama (version 1.7.4), concatenated, and a kNN graph was constructed on the Scanorama-corrected embedding (X_scanorama). UMAP was computed for visualization, and clusters were identified using Leiden clustering (resolution = 0.6). Cluster labels were visualized in UMAP space and projected onto tissue sections using sc.pl.spatial.

Signature scoring and visualization were additionally performed in Seurat to match common Visium workflows. In Seurat, UMI counts were log-normalized (scale factor = 10,000). To map the spatial distribution of *SLC6A20*+ epithelial cells and *SPP1*+ TAMs, we constructed scRNA-seq-derived gene signatures (top 30 specifically expressed genes) and computed spot-level signature scores with AddModuleScore from the normalized expression matrix [17]. Signature score maps were visualized using SpatialFeaturePlot across sections C2–C4. Spatial co-enrichment of the two programs was quantified within each section using Spearman correlation between spot-level *SLC6A20*+ epithelial cells and *SPP1*+ TAMs signature scores.

As an orthogonal validation, we used Tangram (v1.0.4) in Scanpy to map our integrated, annotated scRNA-seq reference dataset to Visium spots [18]. Marker genes for each subset were identified with rank_genes_groups, and the top 100 genes per subset were used as training genes for Tangram. Tangram-derived spot-level subset probabilities were visualized as normalized spatial annotation maps and compared with signature score maps to evaluate concordance of regional enrichment.

To assess metabolic programs in situ, we calculated spot-level module scores for vitamin B6 metabolism and phenylalanine, tyrosine, and tryptophan biosynthesis using AddModuleScore with the corresponding pathway gene sets, and visualized these scores with SpatialFeaturePlot, focusing on regions co-enriched for the *SLC6A20*+ epithelial cells and *SPP1*+ TAMs programs.

To support predicted cell–cell communication, we examined spatial expression of six candidate ligand-receptor pairs in section C2 (COL6A1-ITGB1, SPON2-ITGB2, AREG-MMP9, LTB-TNFRSF1A, TFPI-LRP1, LGALS3-ANXA2). Ligand and receptor expression were visualized spatially, and spot-level Spearman correlations were computed between the epithelial signature score and ligand (or receptor) expression, as well as between the TAM signature score and the corresponding receptor (or ligand) expression, as hypothesis-generating in situ evidence for coordinated ligand–receptor expression.

### 2.13. Targeted Metabolite Analysis

Targeted metabolomics data from MTBLS8090, including bulk colorectal cancer and control samples, were analyzed. These bulk metabolomics data were used as tissue-level support for tumor-associated metabolic alterations, rather than as cell type-specific or spatially resolved validation. Metabolite intensities were extracted from the study matrices, with missing values coded as 9.0, treated as absent signals, and imputed using half of the minimum observed value for each metabolite. After median normalization and log2 transformation, metabolites related to aromatic amino acid metabolism and vitamin B6 metabolism were selected a priori based on biological relevance. Differential abundance between tumor and control samples was evaluated using limma (version 3.58.1) with empirical Bayes moderation. For metabolites represented by more than one analytical feature, the result with the strongest statistical support was retained. *p*-values were further adjusted within the targeted metabolite panel using the Benjamini–Hochberg method, and metabolites meeting raw *p* < 0.05 or panel-adjusted *p* < 0.10 were included for visualization.

### 2.14. Identification of Differentially Expressed Genes, Clustering, and Hub Gene Identification

To identify subtype-specific highly expressed genes for *SPP1*+ TAMs and *SLC6A20*+ epithelial cells, we used the sc.tl.rank_genes_groups function in Scanpy to perform differential gene expression analysis with the Wilcoxon test. Genes with a *p*-value < 0.05 and log2 fold change > 1.0 were selected, ranked by *p*-value and log2 fold change, and the subtype-specific highly expressed genes for both clusters were identified. We selected the top 20 specific highly expressed genes for both *SPP1*+ TAMs and *SLC6A20*+ epithelial cells. Subsequently, using these 40 genes, we performed consensus clustering with the ConsensusClusterPlus (version 1.66.0) method, utilizing 500 iterations and 80% resampling based on the TCGA-COAD dataset, and the clustering resulted in two distinct clusters, C1 and C2.

The DESeq2 (version 1.42.0) and edgeR (version 4.0.16) were used to calculate the differentially expressed genes (DEGs) between the C1 and C2 subtypes, and genes with absolute log2 fold change > 1 and Benjamini–Hochberg adjusted *p*-value < 0.05 were regarded as DEGs. The common DEGs identified by both DESeq2 and edgeR were assigned higher weights and submitted to the STRING website to retrieve the protein–protein interaction (PPI) network. Visualization and hub genes identification were implemented inside Cytoscape (version 3.10.3) using the cytoHubba (version 0.1) plugin.

### 2.15. Construction of a Prognostic Model for High-Risk Classification

In order to train multivariable statistical models for predicting the high-risk subtype, a total of 447 TCGA tumor samples with sufficient survival information were used for model development. About 313 (70%) samples were selected randomly for the training dataset and the remaining 134 (30%) samples were selected for the test dataset. Using the R package glmnet (version 4.1.8), the elastic net fitting (α = 0.95, λ = 0.01 and 10-fold cross-validation) was implemented to perform a penalized multiple logistic regression on all hub genes simultaneously to identify predictive genes.

The Prediction-Score was calculated as a weighted linear combination of the selected genes as follows:

Prediction-Score = 0.469 × *MARCO* + 0.056 × *TNC* + 0.043 × *MMP9* + (−0.090) × *SFRP1* + 0.127 × *COMP* + 0.179 × *SPP1* + 0.022 × *POSTN* + 0.045 × *ITGA11* + 0.511 × *SFRP2* + 0.231 × *WNT7A* + (−0.034) × *CD163* + 0.032 × *KRT14* + 0.218 × *SERPINB2* + (−0.119) × *FCGR3A* − 13.132

The cutoff was derived from the TCGA training cohort and applied unchanged to the TCGA test cohort and external validation cohorts, including GSE38832, GSE39582, and GSE17538. Patients with scores above the cutoff were classified as predicted high-risk. Model performance was assessed by ROC analysis, and survival differences were evaluated using Kaplan–Meier analysis with log-rank tests.

### 2.16. Survival Analysis

We calculated cell infiltration scores for epithelial (*EPCAM*, *KRT19*, *KRT18*) and myeloid (*FCGR3A*, *CD14*, *C1QA*) markers in the TCGA-COAD dataset, dividing samples into high-infiltration and low-infiltration groups based on the median score. Survival differences between the ‘BothHigh’ group (high infiltration of both cell types) and other groups were assessed using the survival package (version 3.4.0). Optimal cutoff points for both scores were determined with the cutoff::logrank method from the cutoff package (version 1.3) and refined using the surv_cutpoint function from the survminer package (version 0.5.0). Kaplan–Meier survival curves were plotted for both dual and individual cell infiltrations.

The elastic net model was then applied to predict risk scores for patients in the TCGA test dataset and the GSE38832, GSE39582, and GSE17538 datasets, classifying samples into high-risk and low-risk groups. Survival differences between these groups were evaluated with Kaplan–Meier curves and the log-rank test.

### 2.17. Statistical Analysis

All statistical analyses and visualizations were performed using R software (version 4.2.2) and Python (version 3.12.2). Differences in cell proportions between the two tissue types were assessed using the independent two-sample *t*-test, which was conducted in Python. The same statistical test was applied to compare the distributions of cell scores between the two tissue types. The Wilcoxon signed-rank test, also performed in Python, was used to compare the percentage of gene-positive cells between Normal and Tumor tissues within each patient. For enrichment analyses, conducted in R, multiple testing correction was performed using the Benjamini–Hochberg method.

## 3. Results

### 3.1. Integration of Single-Cell Transcriptomic Datasets Reveals a Comprehensive Cellular Landscape of Colorectal Cancer

**Figure 1:**
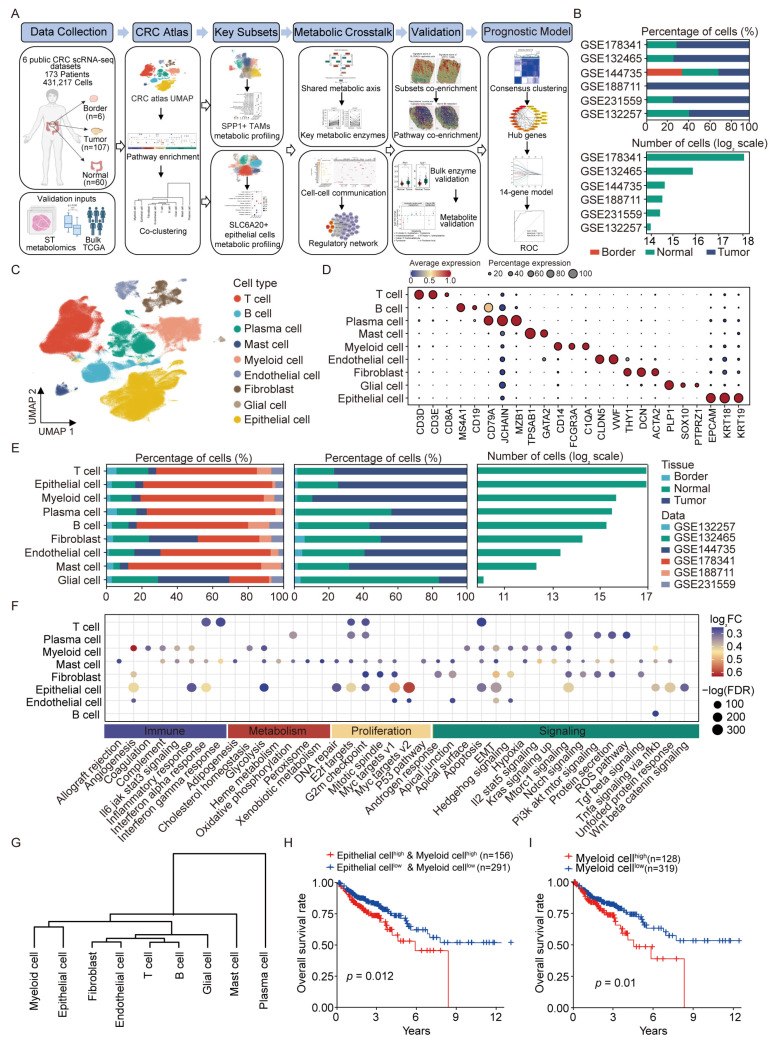
TME of CRC at single-cell resolution. (**A**) Graphic overview of this study design. Arrows indicate the sequential analytical workflow. (**B**) Proportion of tissue types across the six single-cell CRC datasets and total cell number in each dataset are shown as bar plots. (**C**) The Uniform Manifold Approximation and Projection (UMAP) plot of all main cell types. (**D**) Dot plots showing average expression of known markers in indicated cell clusters. Dot size represents the percentage of cells expressing each gene in each cluster, and color intensity indicates the average expression level. (**E**) Bar plots showing the proportions of the nine major cell types across different datasets (**left**), tissues (**middle**), and the total number of each cell type (**right**). (**F**) Dot plots of 40 hallmarks for differentially expressed genes in the global cell types between tumor and normal tissues. The intensity represents the average log2 fold change (log2FC) of gene expression in tumor versus normal mucosa. Dot size shows −log(FDR) for each hallmark. Wilcoxon signed-rank test was used to assess the difference. ROS, Reactive Oxygen Species pathway; EMT, Epithelial–Mesenchymal Transition. (**G**) Hierarchical clustering dendrogram of nine cell subpopulations based on their mean gene expression profiles. (**H**,**I**) Kaplan–Meier plots showing worse clinical outcomes in patients with high infiltration of myeloid cells and epithelial cells (**H**) and in patients with high infiltration of myeloid cells alone (**I**).

We integrated six publicly available CRC single-cell RNA sequencing (scRNA-seq) datasets—GSE178341 [19], GSE132465, GSE144735, GSE132257 [20], GSE188711 [21], and GSE231559 [22]—to construct a unified atlas comprising 431,217 high-quality cells from 173 patients (303,202 tumor, 9069 border, 118,946 normal; Figure 1A,B and Figure S1A–C). Batch effects were effectively minimized through scVI and scANVI integration methods [23], resulting in well-mixed Uniform Manifold Approximation and Projection (UMAP) embeddings (Figure 1C). Annotation based on canonical markers identified nine major cell types (Figure 1C,D), with T cells and epithelial cells constituting the largest fractions. Glial cells were rare and predominantly derived from adjacent normal mucosa (Figure 1E). Epithelial cells displayed marked intratumoral heterogeneity, while both fibroblast and epithelial subpopulations showed clear segregation based on tissue origin, indicating tumor-associated transcriptional reprogramming (Figure 1C,E and Figure S1B). Quantitative comparisons revealed significant enrichment of T cells, epithelial cells, and myeloid cells in tumors relative to normal tissue (Figure 1E), consistent with previous observations in CRC [24,25].

Pathway enrichment analysis was performed using MSigDB hallmark gene sets 15, comparing tumor and normal tissue-derived cells across eight major cell types (excluding glial cells due to limited numbers; Figure 1F). This revealed upregulation of cholesterol homeostasis, glycolysis, and oxidative phosphorylation in tumor-derived plasma cells, myeloid cells, and epithelial cells. Notably, glycolysis was prominently elevated in tumor myeloid and epithelial cells, consistent with known aerobic glycolysis in cancer and lactate-mediated immunosuppression by myeloid cells [26,27].

Clustering of cell subpopulations based on average gene expression highlighted a strong myeloid-epithelial co-clustering pattern (Figure 1G). Patients exhibiting high infiltration of both lineages demonstrated significantly poorer survival outcomes, while only epithelial cells showed no survival difference (Figure 1H,I and Figure S1D). These findings suggest that metabolic complementarity between tumor-associated epithelial and myeloid cells may facilitate CRC progression and contribute to adverse prognosis.

### 3.2. Tumor-Enriched SPP1+ Macrophages Exhibit Immunosuppressive Polarization and Terminal-like Differentiation Features

**Figure 2:**
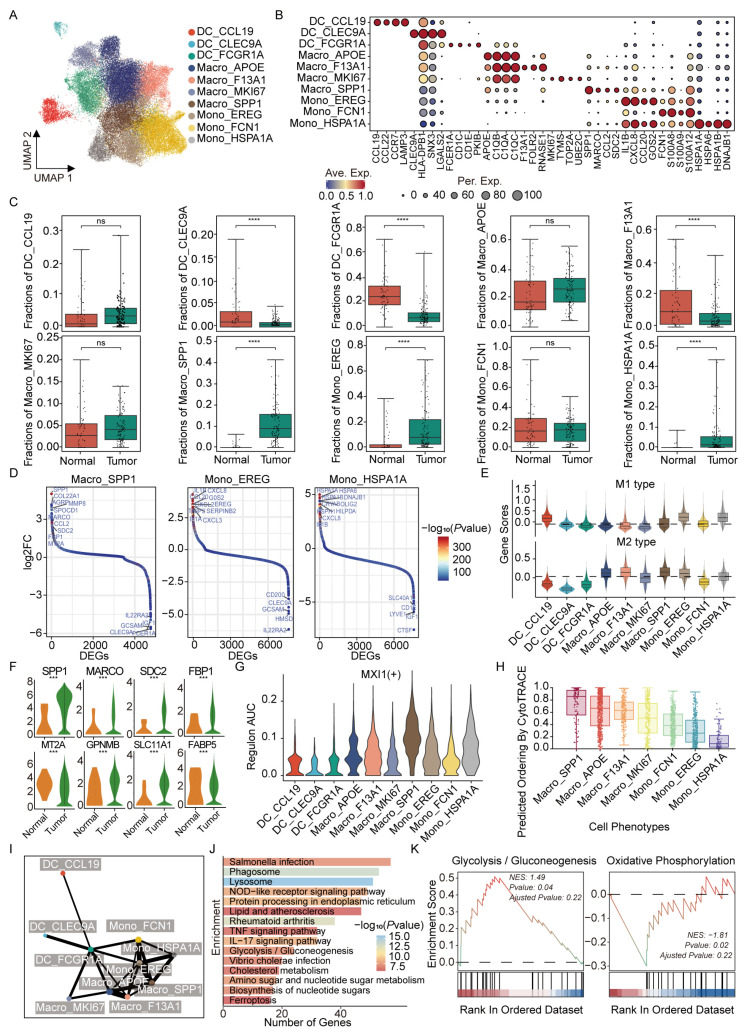
Characterization of myeloid cells in normal mucosa and tumor tissue. (**A**) UMAP of individual myeloid cells. (**B**) Expression levels and frequencies of selected markers across myeloid subtypes. (**C**) Comparison of the proportions of each myeloid subtype between normal and tumor. ns, not significant; ****, *p* < 0.0001. *p*-values were determined by the Wilcoxon test. (**D**) Ranked differentially expressed genes (DEGs) in Mono_EREG, Mono_HSPA1A, and Macro_SPP1. (**E**) Expression levels of M1 and M2 signatures across myeloid subtypes. (**F**) Expression of selected genes in *SPP1*+ M2-like macrophages in tumor and normal tissues. ***, *p* < 0.001. (**G**) Violin plot showing the distribution of MXI1 regulon activity (AUCell AUC scores) across myeloid cell subclusters. (**H**) CytoTRACE analysis of macrophage and monocyte subtypes. (**I**) PAGA pseudospatial trajectory analysis of myeloid subtypes. (**J**) KEGG enrichment analysis of the top 15 upregulated pathways in Macro_SPP1. (**K**) GSEA of metabolic pathways enriched in Macro_SPP1.

Given the profound metabolic rewiring observed in tumor epithelial and myeloid cells and their frequent co-occurrence within the CRC microenvironment, we next dissected myeloid heterogeneity. Unsupervised re-clustering and marker-based annotation delineated ten transcriptionally distinct myeloid subsets (Figure 2A,B), including three dendritic cell (DC) populations (DC_CCL19, DC_CLEC9A, DC_FCGR1A), three monocyte subsets (Mono_EREG, Mono_FCN1, Mono_HSPA1A), and four macrophage subsets (Macro_APOE, Macro_F13A1, Macro_MKI67, Macro_SPP1). Quantitative comparison revealed tumor enrichment of Macro_SPP1, Mono_HSPA1A, and Mono_EREG, whereas DC_CLEC9A, DC_FCGR1A, and Macro_F13A1 predominated in normal tissues (Figure 2C), indicating tumor-associated myeloid reprogramming.

Polarization profiling showed Mono_EREG and Mono_HSPA1A expressing inflammatory mediators (*CXCL8*, *CCL20*, *IL1B*) with elevated M1 scores, consistent with pro-inflammatory, anti-tumor functions (Figure 2D–F and Figure S2A–D) [28]. In contrast, Macro_SPP1 cells highly expressed *SPP1*, *APOE*, and *APOC1*, and exhibited the highest M2-associated signature score, suggesting an M2-like immunosuppressive and tumor-promoting transcriptional program. (Figure 2D,F) [29]. SPP1 was highly specific and specifically expressed in myeloid cells (Figure S2E). In the integrated scRNA-seq dataset and three independent cohorts (GSE38832, GSE39582, and GSE17538), SPP1 expression showed significant positive correlations with ssGSEA pathway scores for TGF-β signaling and the IFN-α response (Figure S2F–I), which supports the immunosuppressive phenotype of Macro_SPP1 [30]. Transcription factor analysis identified *MXI1* as a potential upstream regulator driving this polarization and specialization (Figure 2G and Figure S2J) [31,32]. Trajectory inference suggested progressive loss of stem-like features from monocytes to macrophages, with Macro_SPP1 showing the lowest stemness score among macrophage subsets (Figure 2H). PAGA analysis further placed Macro_SPP1 at the end of a computationally inferred trajectory branch, consistent with a putative late-differentiation macrophage state characterized by mature macrophage transcriptional programs (Figure 2I). KEGG and GSEA analyses showed Macro_SPP1 cells enriched for glycolysis/gluconeogenesis, cholesterol metabolism, and nucleotide sugar biosynthesis pathways, coupled with downregulated oxidative phosphorylation (Figure 2J,K), indicating a metabolic shift favoring glycolytic and lipid metabolism. Such metabolic remodeling was absent in Mono_EREG and Mono_HSPA1A subsets (Figure S2A–D), highlighting the unique metabolic phenotype of Macro_SPP1. Collectively, these data suggest that tumor-associated macrophage Macro_SPP1 (*SPP1*+ TAMs) represent a metabolically reprogrammed and immunosuppressive macrophage population with terminal-like differentiation features, which may shape the CRC immune microenvironment and contribute to tumor progression.

### 3.3. Distinct Metabolic Rewiring Characterizes SPP1+ TAMs

**Figure 3:**
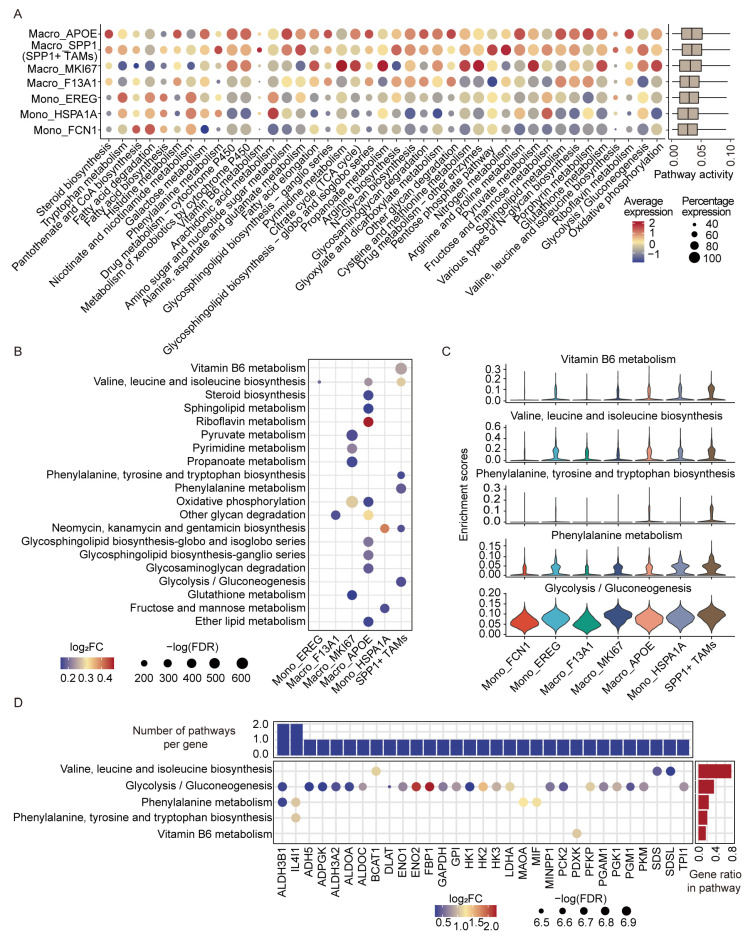
*SPP1*+ TAMs show high metabolic activity. (**A**) Metabolic activity analysis revealed that Macro_APOE and Macro_SPP1 (*SPP1*+ TAMs) had the highest metabolic scores. (**B**) Dot plot of metabolic pathway enrichment across myeloid subtypes. (**C**) Enrichment of five highly expressed metabolic pathways in *SPP1*+ TAMs across myeloid subtypes. (**D**) Distribution of differentially expressed genes (DEGs) from the *SPP1*+ TAMs across five enriched KEGG metabolic pathways. The top bar chart depicts the number of enriched pathways per DEG, ordered by descending frequency. The central dot plot maps each DEG (x-axis) to the five selected pathways (y-axis). The right bar chart represents the DEG-to-total gene ratio for each pathway.

To comprehensively characterize the metabolic landscape of myeloid populations in CRC, we quantified the activity of 82 canonical metabolic pathways at single-cell resolution. Ranking pathway activity within the top 50th percentile identified that *SPP1*+ TAMs and Macro_APOE exhibited the highest overall metabolic activity among tumor-infiltrating myeloid cells (Figure 3A and Figure S3A), indicating pronounced metabolic reprogramming.

Comparative pathway analysis revealed a unique metabolic signature in *SPP1*+ TAMs, marked by selective upregulation of vitamin B6 metabolism, branched-chain amino acid biosynthesis (valine, leucine, isoleucine), aromatic amino acid biosynthesis and metabolism (phenylalanine, tyrosine, tryptophan), glycolysis/gluconeogenesis, and antibiotic-related biosynthesis (Figure 3B,C). This metabolic profile suggests a coordinated metabolic pattern coupling enhanced glycolytic flux to increased amino acid and vitamin metabolism, which may modulate immune responses and reinforce tumor-immune interactions.

Intersecting *SPP1*+ TAMs specific differentially expressed genes with those involved in these metabolic pathways identified 31 metabolism-related candidate effectors (Figure 3D and Figure S3B). Notably, IL4I1, an aromatic amino acid oxidase, generates aryl hydrocarbon receptor (AhR) ligands that promote tumor cell migration and suppress T cell activation [33]. PDXK, a key kinase in vitamin B6 metabolism, catalyzes phosphorylation of pyridoxal to pyridoxal 5′-phosphate, facilitating amino acid metabolism, glutathione biosynthesis, and oxidative stress responses, potentially fostering an immunosuppressive milieu [34,35]. These findings delineate a specialized metabolic program in *SPP1*+ macrophages that likely contributes to immune modulation and CRC progression.

### 3.4. Highly Metabolically Active Epi01 Malignant Epithelial Subcluster Reflects Tumor Heterogeneity in CRC

**Figure 4:**
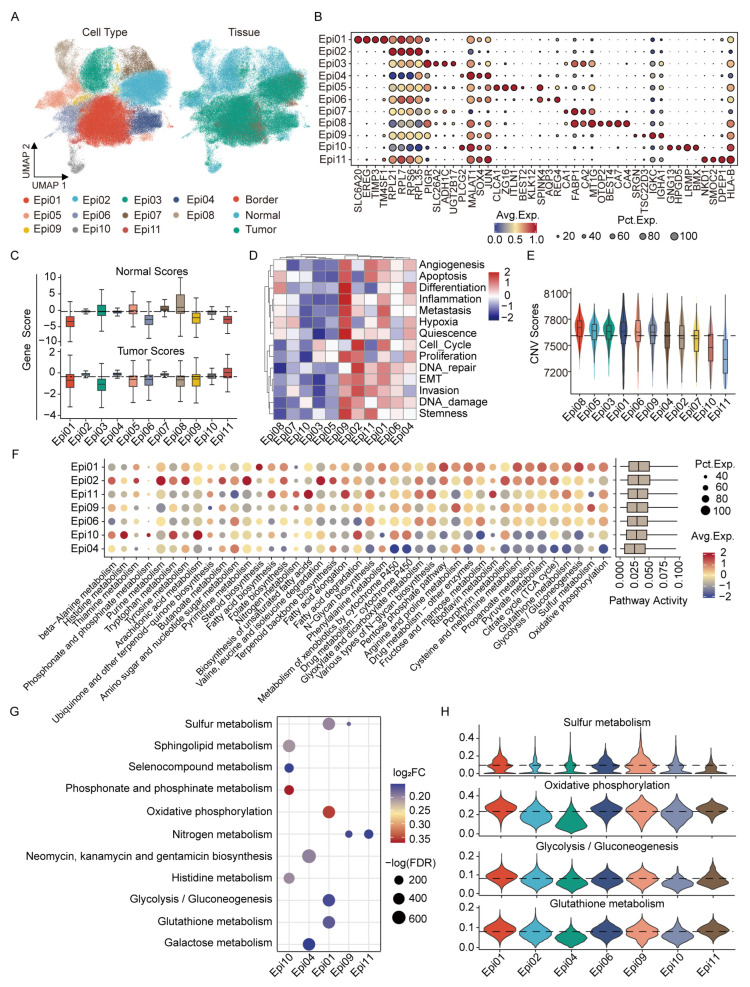
Characterization of epithelial cells in normal mucosa and tumor tissue. (**A**) UMAP showing the composition of epithelial cells colored by cluster and class. (**B**) Expression levels and frequencies of selected markers across epithelial subtypes. (**C**) Boxplots of module scores across epithelial subtypes, based on gene signatures derived from TCGA-COAD ‘normal’ and ‘tumor’ cohorts. The upper panel shows scores for the normal signature; the lower panel shows scores for the tumor signature. (**D**) Heatmap shows functional-state gene set scores for each epithelial subtype as assessed by CancerSEA. (**E**) Violin plot showing the copy number variation (CNV) scores of each epithelial subtype. (**F**) Metabolic activity analysis revealed that Epi01 had the highest metabolic score. Circle size and color intensity both represent the scaled metabolic score. (**G**) Dot plot of metabolic pathway enrichment across tumor epithelial subtypes. Dot size represents −log(FDR), and color indicates log2 fold change (log2FC). (**H**) Enrichment of four highly expressed metabolic pathways in Epi01 across epithelial subtypes.

Tumor epithelial cells drive CRC progression and show marked metabolic heterogeneity that can influence proliferation, invasion, and immune modulation via metabolite secretion and nutrient competition [36]. To characterize epithelial metabolic remodeling, we re-clustered epithelial cells and identified 11 metabolically distinct subpopulations spanning normal, tumor, and border origins (Figure 4A,B). Module score analysis leveraging tumor-specific and normal-specific gene signatures from the TCGA-COAD cohort classified Epi03/05/07/08 as normal-like, Epi01/02/04/06/09/11 as tumor-like, and Epi10 as transitional (Figure 4C and Figure S4A,B). Functional profiling with CancerSEA’s 14 cell state gene sets partitioned epithelial clusters into low-activity (Epi03, Epi05, Epi07, Epi08, Epi10) and high-activity groups (Epi01, Epi02, Epi04, Epi06, Epi09, Epi11), the latter enriched for metastasis, proliferation, EMT, and DNA repair signatures (Figure 4D). InferCNV analysis revealed increased copy number variation (CNV) burden across tumor-like clusters, with Epi01 exhibiting the highest genomic instability (Figure 4E).

Further metabolic pathway scoring across 82 KEGG sets within the top 50th percentile ranked Epi01 as the most metabolically reprogrammed malignant subset (Figure 4F), characterized by selective activation of glutathione metabolism, glycolysis/gluconeogenesis, oxidative phosphorylation, and sulfur metabolism pathways (Figure 4G,H). These adaptations may collectively fulfill elevated bioenergetic and redox demands within the tumor microenvironment.

Taken together, these data establish Epi01 as the predominant metabolically reprogrammed malignant epithelial subcluster in CRC, marked by enhanced glycolysis and genomic instability. Importantly, this metabolic phenotype closely parallels that of immunosuppressive *SPP1*+ macrophages, suggesting a functional metabolic crosstalk that merits further investigation into ligand–receptor interactions and additional metabolic pathways mediating their reciprocal communication.

### 3.5. Cooperative Metabolic Reprogramming Between SPP1+ TAMs and SLC6A20+ Epithelial Cells Is Associated with the Tumor Microenvironment Remodeling

**Figure 5:**
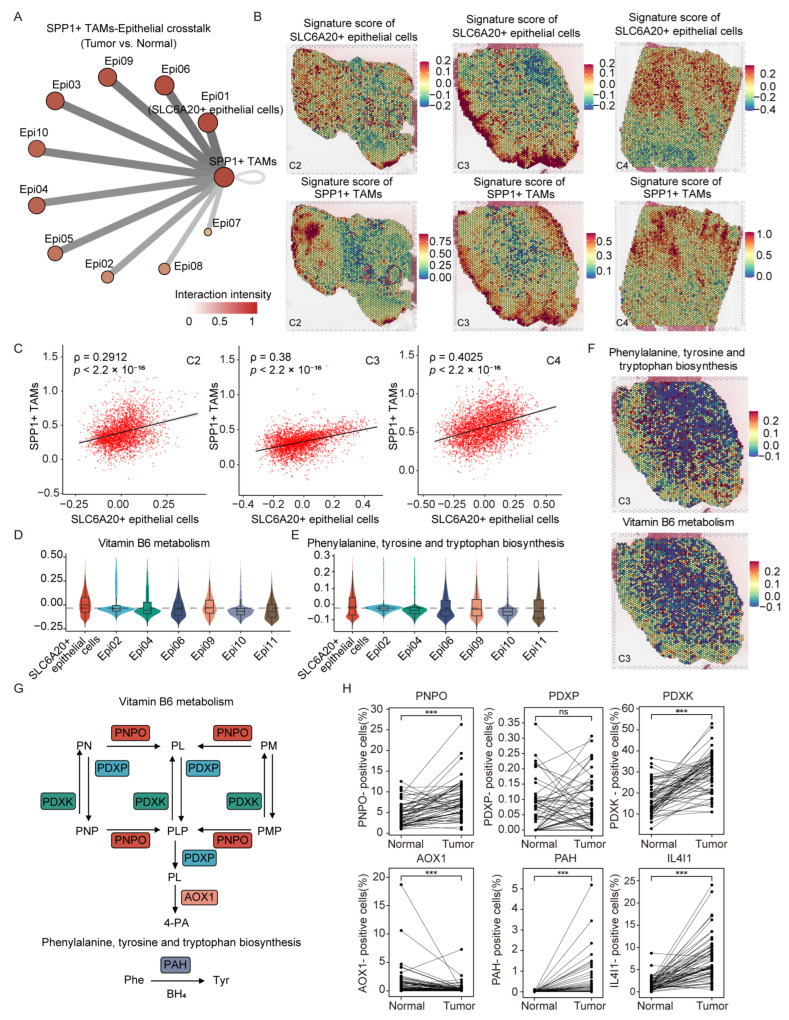
Putative metabolic crosstalk between *SPP1*+ TAMs and *SLC6A20*+ epithelial cells via vitamin B6 and aromatic amino acid pathways. (**A**) Ranked differential crosstalk between *SPP1*+ TAMs and epithelial subsets (tumor vs. normal) shows that Epi01 (*SLC6A20*+ epithelial cells) ranks first among all epithelial subsets. (**B**) Spatial feature plots showing signature scores of *SLC6A20*+ epithelial cells and *SPP1*+ TAMs in tissue sections from C2–C4. (**C**) Spearman correlation between spot-level signature scores of *SLC6A20*+ epithelial cells and *SPP1*+ TAMs. (**D**,**E**) Violin plot of vitamin B6 metabolism (**D**) and phenylalanine tyrosine tryptophan biosynthesis (**E**) module scores across tumor epithelial subclusters. (**F**) Spatial feature plots showing pathway activity scores of vitamin B6 metabolism and phenylalanine tyrosine tryptophan biosynthesis in C3. (**G**) Schematic diagram of human vitamin B6 metabolism and phenylalanine tyrosine tryptophan biosynthesis pathways. (**H**) Paired line plots of the percentage of epithelial cells positive for *PNPO*, *PDXP*, *PDXK*, *AOX1*, *PAH* and *IL4I1* in matched normal and tumor samples (n = 57). ***, *p* < 0.001; ns, not significant (*p* ≥ 0.05).

Metabolic overlap between tumor cells and immune infiltrates is a critical determinant of antitumor immunity [37]. Given the pro-tumoral activity of M2-polarized *SPP1*+ TAMs, we hypothesized a metabolic co-regulation with a distinct tumor epithelial subset. Ligand–receptor interaction analysis revealed Epi01 (*SLC6A20*+ epithelial cells) as the epithelial cluster exhibiting the strongest interaction score with *SPP1*+ TAMs among all tumor-derived epithelial populations (Figure 5A). This suggests that *SLC6A20*+ epithelial cells may be associated with metabolic reprogramming and immunosuppression in tumor-associated macrophages via ligand-mediated signaling. To substantiate this inference in the ST datasets GSE225857 [38], scoring each spot with *SLC6A20*+ epithelial cells and *SPP1*+ TAMs signatures derived from scRNA-seq data (top 30 specifically expressed genes) supported regional co-enrichment of the two signatures within the same tumor regions (Figure 5B and Figure S5A). This regional co-enrichment pattern was further supported by Tangram mapping and Cell2location-based deconvolution (Figure S5B,C). In addition, the signature score of *SLC6A20*+ epithelial cells and *SPP1*+ TAMs showed a significantly positive correlation (Figure 5C).

We quantified activity of five metabolic pathways enriched in *SPP1*+ TAMs across epithelial clusters and observed significantly elevated vitamin B6 metabolism and aromatic amino acid biosynthesis activity in *SLC6A20*+ epithelial cells relative to other subpopulations (Figure 5D,E and Figure S5D–J), highlighting their central roles in the metabolic interplay between these two cell types. In spatial transcriptomics, vitamin B6 and aromatic amino acid module scores were also elevated in regions co-enriched for *SLC6A20*+ epithelial and *SPP1*+ TAM signatures (Figure 5F and Figure S5K). Vitamin B6 metabolism involves PDXK-mediated phosphorylation of vitamin B6 vitamers to their active 5′-phosphate forms, PNPO-driven oxidation to the coenzyme pyridoxal phosphate (PLP), PDXP-mediated dephosphorylation and PLP regeneration, and AOX1-catalyzed oxidation for vitamin B6 clearance [39]. In aromatic amino acid metabolism, human cells retain only PAH-catalyzed phenylalanine to tyrosine conversion (Figure 5G), while IL4I1 acts as an aromatic amino acid oxidase catabolizing phenylalanine and tryptophan to immunomodulatory metabolites [40,41].

In paired normal-tumor samples, *PDXK*, *PNPO*, *PAH*, and *IL4I1* were significantly upregulated in tumors (*p* < 0.05), whereas *PDXP* was unchanged and *AOX1* was higher in normal tissue (Figure 5H). This tumor-associated expression pattern was further validated in two independent bulk CRC cohorts, in which *PNPO*, *PDXK*, *PAH*, and *IL4I1* were likewise significantly upregulated in tumors in both GSE44076 and GSE113513 (Figure S5L,M). Analysis of the independent targeted metabolomics dataset MTBLS8090 [42] additionally identified tumor-associated alterations in metabolites linked to aromatic amino acid metabolism and vitamin B6 metabolism, providing bulk tissue-level metabolite support for dysregulation of these two metabolic pathways in tumors (Figure S5N). Notably, *PDXK* expression correlates with poor prognosis and Wnt/β-catenin activation, *PNPO* is associated with immune suppression across solid tumors [43], PAH dysregulation has been reported in CRC metabolomics [44], and *IL4I1* promotes immune evasion via AhR pathway activation [41].

Together, coordinated regulation of vitamin B6 and aromatic amino acid metabolic pathways within the *SPP1*+ TAMs − *SLC6A20*+ epithelial cells axis suggests potential molecular mechanisms underlying metabolic adaptation and immune tolerance in CRC, and may provide candidate targets for combined metabolic immunotherapeutic strategies.

### 3.6. Reciprocal Signaling Between SPP1+ TAMs and SLC6A20+ Epithelial Cells Is Associated with Metabolic and Proliferative Remodeling in CRC

To elucidate the molecular basis underlying crosstalk between *SPP1*+ TAMs and *SLC6A20*+ epithelial cells in CRC, we applied NicheNet analysis to infer ligand–receptor interactions and downstream regulatory programs. Our results suggest that *SLC6A20*+ epithelial cells may engage *SPP1*+ TAMs via adhesive interactions, including COL6A1 binding to integrins ITGB1 and ITGA6 (Figure S6A). ST analysis indicated spatial co-enrichment of COL6A1-ITGB1, SPON2-ITGB2, and AREG-MMP9 with *SLC6A20*+ epithelial cells and *SPP1*+ TAMs (Figure S6B). Elevated expression of BACE2 in *SLC6A20*+ epithelial cells may enhance β-secretase cleavage of APP, generating metabolites that have been previously shown to inhibit mitochondrial oxidative phosphorylation and promote glycolytic flux in recipient cells [45,46]. Additionally, *SLC6A20*+ epithelial cells secrete SPON2, which interacts with integrins ITGB1, ITGAM, and ITGB2 on *SPP1*+ TAMs (Figure S6C), potentially activating FAK-PI3K-AKT-mTOR signaling cascades that support M2-like macrophage polarization [47]. This signaling upregulates lipid-handling proteins such as FABP4, enhancing fatty acid uptake and lipid droplet accumulation [48]. The resulting lipid-rich phenotype correlates with elevated expression of immunosuppressive markers ARG1, VEGFα, and IL-10 [49], reinforcing the tumor-promoting function of *SPP1*+ TAMs.

**Figure 6:**
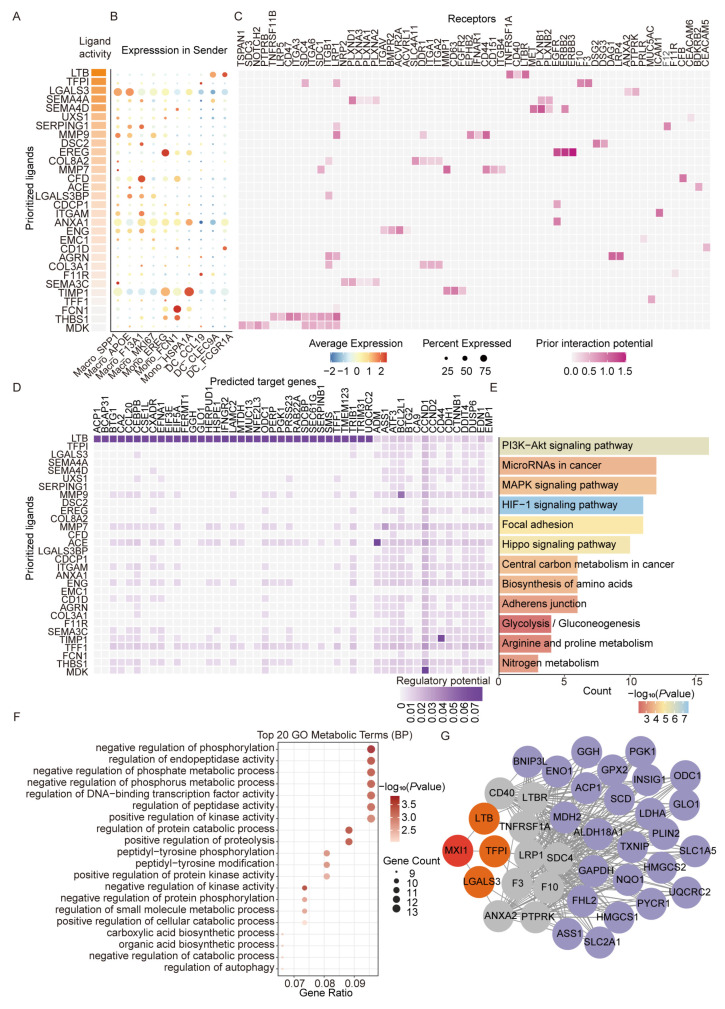
Predicted interaction network between *SPP1*+ TAMs and *SLC6A20*+ epithelial cells. (**A**) Top-ranked ligands inferred to regulate *SLC6A20*+ epithelial cells by *SPP1*+ TAMs according to NicheNet. (**B**) Dot plots showing the expression percentage (dot size) and intensity (color intensity) of top-ranked ligands in each myeloid subtype. (**C**) Ligand–receptor pairs predicted to mediate communication between *SPP1*+ TAMs and *SLC6A20*+ epithelial cells ordered by ligand activity. (**D**) Heatmap showing regulatory potential of top 30 ranked ligands and the downstream target genes in *SLC6A20*+ epithelial cells. (**E**) Representative KEGG signaling and metabolic pathway enrichment of the predicted target genes expressed in *SLC6A20*+ epithelial cells. (**F**) Dot plots show the GO metabolic pathways enriched by the downstream target genes expressed in *SLC6A20*+ epithelial cells. BP, Biological Process. (**G**) Cross-cell regulatory network from *SPP1*+ TAMs to *SLC6A20*+ epithelial cells: predicted transcription factors and candidate ligands of the *SPP1*+ TAMs, receptors expressed by the *SLC6A20*+ epithelial cells, and metabolism-related downstream target genes.

Given this metabolic-immune interplay, we further investigated whether *SPP1*+ TAMs may reciprocally modulate *SLC6A20*+ epithelial cells. NicheNet analysis identified macrophage-derived ligands LTB, TFPI, and LGALS3 as candidate ligands with predicted regulatory potential toward *SLC6A20*+ epithelial cells. (Figure 6A–C). LTB binds receptors TNFRSF1A, CD40, and LTBR; TFPI interacts with SDC4, LRP1, F10, and F3, while LGALS3 engages ANXA2 and PTPRK on *SLC6A20*+ epithelial cells. ST analysis showed that ligand expression (LTB, TFPI, LGALS3) co-enriched with *SPP1*+ TAMs signals, whereas receptor expression (TNFRSF1A, LRP1, ANXA2) co-enriched with *SLC6A20*+ epithelial cells signals in C2 (Figure S6D). KEGG pathway enrichment of *SLC6A20*+ epithelial cells downstream targets revealed functional modules associated with proliferation and survival pathways (PI3K/Akt, MAPK, Hippo), adhesion and motility (focal adhesion, adherens junction), and immune regulation (TGF-β, HIF-1 signaling) (Figure 6D,E). Gene Ontology analysis further highlighted metabolic programs including glycolysis, pyruvate metabolism, nucleotide phosphorylation, ketone body turnover, and glutamine amino acid biosynthesis (Figure 6D,F). Collectively, these data suggest that *SPP1*+ TAMs may engage signaling programs associated with increased proliferative capacity, invasive potential, and immunosuppressive phenotypes in *SLC6A20*+ epithelial cells, while coupling these traits to elevated glycolytic and anabolic metabolism.

Finally, we constructed a regulatory network integrating *SPP1*+ TAMs transcription factor MXI1, its predicted ligands (LTB, TFPI, LGALS3), cognate receptors on *SLC6A20*+ epithelial cells, and 25 metabolism-related downstream target genes (Figure 6G). These downstream targets encompass key pathways in glycolysis, pyruvate metabolism, lipid biosynthesis, amino acid metabolism, and redox homeostasis, delineating a coordinated metabolic rewiring within the tumor microenvironment.

### 3.7. Development of a Prognostic Model for the Metabolic Interaction Between SPP1+ TAMs and SLC6A20+ Epithelial Cells

**Figure 7:**
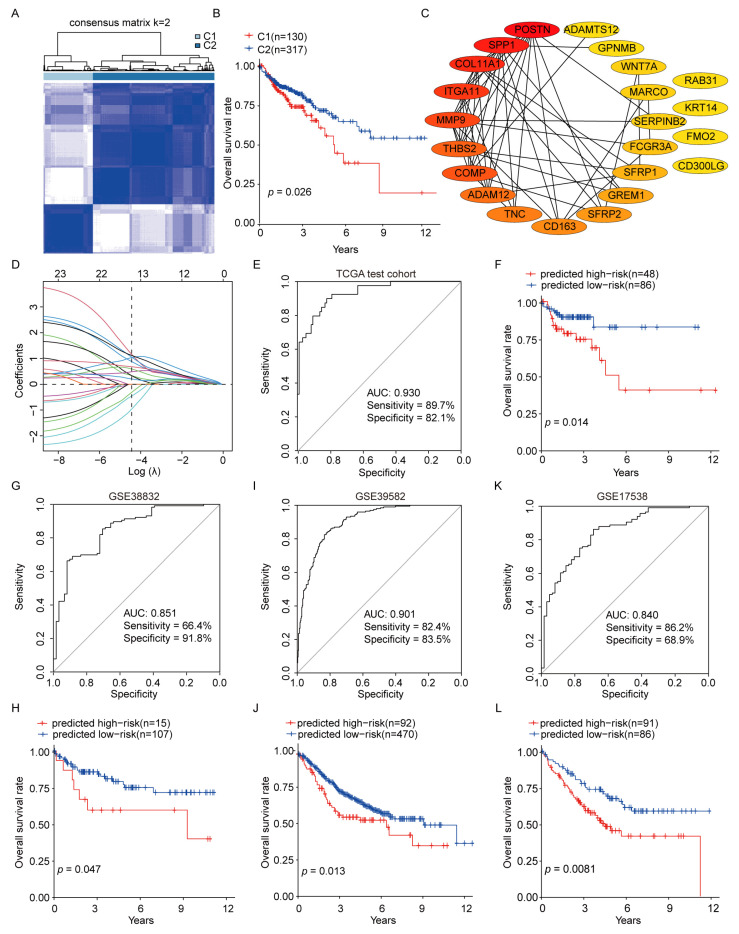
Development of a prognostic model for the high-risk subtype. (**A**) Clustered heatmap of samples based on the top 20 genes from *SPP1*+ TAMs and the top 20 genes from *SLC6A20*+ epithelial cells. (**B**) Kaplan–Meier curve (log-rank test) showing overall survival (OS) for the two clusters (C1 and C2) derived from ConsensusClusterPlus method in the TCGA cohort. (**C**) PPI network of the 23 hub genes. (**D**) Least absolute shrinkage and selection operator (LASSO) analysis of the selected genes. The dashed vertical line indicates the selected λ used for constructing the 14-gene model. (**E**) Receiver operating characteristic (ROC) curves for predictions based on the expression levels of 14 genes in the TCGA test cohort (n = 134). (**F**) Kaplan–Meier curve (log-rank test) showing overall survival (OS) for the predicted high-risk and low-risk subtypes in the TCGA test cohort. (**G**,**I**,**K**) ROC curves for predictions based on the expression levels of 14 genes in the validation cohorts GSE38832 (**G**) (n = 122), GSE39582 (**I**) (n = 562), and GSE17538 (**K**) (n = 177). (**H**,**J**,**L**) Kaplan–Meier curves (log-rank test) showing OS for the predicted high-risk and low-risk subtypes in the validation cohorts GSE38832 (**H**), GSE39582 (**J**), and GSE17538 (**L**).

Acknowledging the important role of *SPP1*+ TAMs and *SLC6A20*+ epithelial cells’ metabolic crosstalk in CRC progression, we investigated their potential as prognostic markers for CRC patients. We performed consensus clustering based on the TCGA-COAD dataset, which resulted in two clusters, C1 and C2 (Figure 7A). The C1 cluster was associated with a poorer prognosis, while the C2 subtype showed a significantly better survival (Figure 7B). Subsequently, we developed a scoring system to accurately predict the molecular subtype (see method Identification of Differentially Expressed Genes, Clustering and Hub Gene Identification). We identified 23 hub genes (*KRT14*, *SFRP1*, *SFRP2*, *SPP1*, *WNT7A*, *MARCO*, *FMO2*, *CD300LG*, *SERPINB2*, *CD163*, *FCGR3A*, *MMP9*, *RAB31*, *GPNMB*, *COMP*, *COL11A1*, *THBS2*, *ADAM12*, *POSTN*, *ADAMTS12*, *ITGA11*, *GREM1* and *TNC*; Figure 7C). Penalized multiple logistic regression analysis was performed on all hub genes, resulting in the selection of 14 predictive genes (*MARCO*, *TNC*, *MMP9*, *SFRP1*, *COMP*, *SPP1*, *POSTN*, *ITGA11*, *SFRP2*, *WNT7A*, *CD163*, *KRT14*, *SERPINB2*, and *FCGR3A*; Figure 7D), which were used to construct the Prediction-Score model.

The performance of the Prediction-Score in predicting subtypes of CRC patients was evaluated. The model achieved significant sensitivity and accuracy on the TCGA test dataset, comprising 134 CRC patients, with an AUC value of 0.930 (Figure 7E). We further tested the model on three other CRC cohorts, including GSE38832, GSE39582, and GSE17538, yielding AUC values of 0.851, 0.901, and 0.840, respectively (Figure 7G,I,K). Moreover, patients with higher Prediction-Scores showed poorer survival across retrospective cohorts (Figure 7F,H,J,L). In GSE39582, multivariate Cox analysis showed that the Prediction-Score remained significantly associated with overall survival after adjustment for age, sex, and TNM stage, but this association was attenuated after further adjustment for CMS subtype (Figure S7A,B). These findings indicate that the Prediction-Score can stratify CRC patients by prognosis, although its independent prognostic value requires further validation.

To further address potential overfitting and reduce model complexity, we additionally constructed a more parsimonious 6-gene LASSO classifier using the lambda.1se criterion. The reduced model included *SFRP2*, *SPP1*, *MARCO*, *SERPINB2*, *COMP*, and *TNC* (Figure S7C). This six-gene classifier showed good subtype-discrimination performance in the TCGA test cohort, GSE39582, and GSE17538, with AUC values of 0.944, 0.938, and 0.928, respectively (Figure S7D,F,H). Survival analysis showed significant stratification in GSE39582, whereas the survival differences in the TCGA test cohort and GSE17538 were not statistically significant (Figure S7E,G,I). Thus, the reduced six-gene classifier supports the robustness of subtype-related transcriptional features, while its prognostic utility remains exploratory and requires further validation.

## 4. Discussion

Metabolic reprogramming is a hallmark of colorectal cancer, underpinning tumor growth, proliferation, and immune evasion [50]. Integrating six CRC scRNA-seq datasets, we resolved subpopulation-level metabolic heterogeneity and found *SLC6A20*+ epithelial cells and *SPP1*+ TAMs adopt distinct yet complementary metabolic programs. *SLC6A20*+ epithelial cells are enriched for glycolysis, glutathione synthesis, and oxidative phosphorylation pathways, reflecting the Warburg effect and the bioenergetic demands of rapid proliferation. In parallel, *SPP1*+ TAMs exhibit enhanced glycolysis-related vitamin B6 and aromatic amino acid metabolic programs, suggesting a hypermetabolic and immunosuppressive TAM state [37,51]. While the M1/M2 framework is useful for describing canonical in vitro macrophage polarization, TAMs in CRC are more likely to exist along a context-dependent continuum. Thus, *SPP1*+ TAMs should be viewed not as a simple counterpart of classical M2 macrophages, but as a tumor-shaped TAM state with immunosuppressive, glycolysis-skewed, and terminal-like differentiation features.

The shared metabolic features of *SLC6A20*+ epithelial cells and *SPP1*+ TAMs suggest a cooperative axis associated with CRC progression. *SLC6A20*+ epithelial cells showed enhanced vitamin B6 metabolism, which may support glycolysis-related energy production and antioxidant defense [52], while lactate and other tumor-derived metabolites could promote immunosuppressive TAM polarization [53]. Conversely, *SPP1*+ TAMs may use enzymes such as PAH and IL4I1 to generate aromatic metabolites that suppress T-cell effector function. These coordinated programs are consistent with a self-reinforcing tumor-immune metabolic association that supports epithelial bioenergetic adaptation and macrophage-mediated immunosuppression [54]. Spatial transcriptomics further supported this axis by showing regional co-enrichment of *SLC6A20*+ epithelial and SPP1+ TAM signatures, together with elevated vitamin B6 and aromatic amino acid pathway scores in the same regions. However, the causal direction of these metabolic alterations remains unresolved. Vitamin B6 and aromatic amino acid metabolic programs may either contribute to intercellular crosstalk or emerge as a consequence of sustained tumor-immune interactions. Thus, they should be interpreted as associated metabolic programs within this axis rather than established causal drivers.

Beyond metabolism, NicheNet analysis suggested reciprocal ligand–receptor signaling between *SLC6A20*+ epithelial cells and *SPP1*+ TAMs, potentially linking metabolic remodeling with proliferation and immune suppression. These interactions are computationally inferred and require functional validation. Epithelial-derived SPON2- and COL6A1-integrin signals are plausible candidates given their reported roles in macrophage recruitment, pro-tumoral polarization, and stromal remodeling [55,56,57]. Conversely, *SPP1*+ TAM-derived LTB, TFPI, and LGALS3 were predicted to engage receptors on *SLC6A20*+ epithelial cells, with LGALS3-ANXA2 appearing more plausible and TFPI-LRP1/LTB-associated signaling remaining lower-confidence candidates [58,59,60]. Together, these predicted interactions suggest a signal-driven, metabolism-supported feedback loop that may sustain a pro-tumoral microenvironment. The tumor-promoting role of *SPP1*+ macrophages is further supported by studies in other cancer contexts. In head and neck squamous cell carcinomas, *SPP1*+ macrophages have been implicated in promoting tumor cell proliferation and migration, partly through inflammatory signaling mediated by TNF-α and IL-1β. In addition, *SPP1*+ TAMs have been reported to regulate metastatic colonization through lymph node niche remodeling and *SPP1*-related ligand–receptor interactions [61,62]. In HER2^+^ breast cancer, *SPP1*^+^ macrophages were associated with an immunosuppressive macrophage state, altered T-cell function, and adverse prognosis [63]. Together, these findings support a broader tumor-promoting role of *SPP1*+ TAMs, while suggesting that their downstream effector mechanisms may vary across tumor contexts.

**Figure 8:**
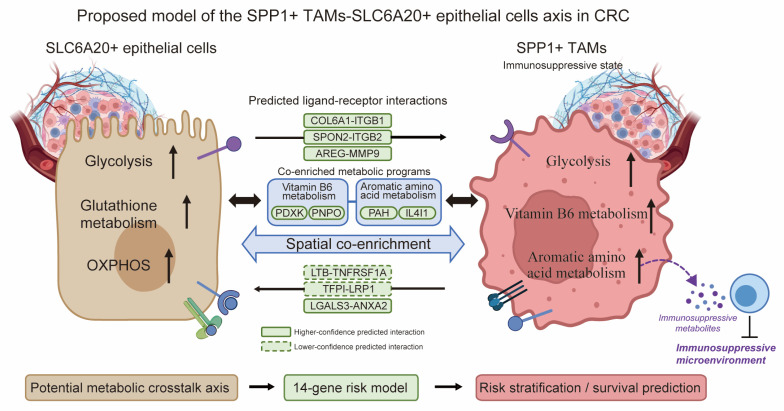
Proposed model of *SPP1*+ TAM-mediated immunosuppressive microenvironment and metabolic crosstalk with *SLC6A20*+ epithelial cells in CRC.

Building on the metabolic interaction features of these two subsets, we constructed a 14-gene elastic net prognostic model that showed robust predictive performance across the test set and independent validation cohorts, suggesting potential relevance for risk stratification. Together, these findings support a proposed working model in which *SPP1*+ TAMs and *SLC6A20*+ epithelial cells form a spatially co-enriched, metabolically rewired, and immunosuppressive tumor microenvironmental axis with prognostic relevance (Figure 8). Beyond prognostic stratification, this axis may help identify a macrophage-rich and metabolically rewired tumor state with potential relevance for biomarker-based patient stratification and rational combination therapy design. Potential macrophage-directed and immunometabolic strategies remain exploratory and require functional validation. In parallel, deep learning has shown increasing potential in CRC digital pathology, including whole-slide histopathology image analysis and MSI/dMMR pre-screening from H&E-stained slides [64,65]. These image-based approaches may improve diagnostic accuracy, efficiency, and reproducibility in clinical pathology. Together with transcriptome-based prognostic models, they may provide complementary information for future CRC risk assessment and patient stratification.

This study is subject to several limitations inherent to its computational and transcriptome-based nature. Our data support an inferred, spatially supported, and biologically plausible association between *SPP1*+ TAMs and *SLC6A20*+ epithelial cells, but do not establish direct causality or a definitive driving role in CRC progression. Metabolic activity and cell–cell interactions were inferred from transcriptomic data, and therefore do not directly measure metabolite abundance, enzymatic activity, or metabolic flux; thus, functional validation using CRC cell–macrophage co-culture, metabolic profiling, qPCR/Western blotting, and perturbation of key mediators such as PDXK and IL4I1 is warranted. Although targeted metabolomics provided orthogonal support, these bulk tissue data were not cell-type specific or spatially resolved. Additionally, residual heterogeneity across public datasets and the retrospective nature of the prognostic model validation should be considered when interpreting the clinical implications. Therefore, the Prediction-Score should be regarded as an exploratory prognostic signature rather than a clinically deployable tool, as its reproducibility may be affected by sequencing platforms, normalization strategies, cohort composition, and preprocessing procedures. Future studies combining single-cell metabolomics, spatial multiomics, and prospective clinical cohorts will be critical to validate and extend these observations.

## 5. Conclusions

In conclusion, our multi-cohort single-cell analysis links *SLC6A20*+ epithelial cells with *SPP1*+ TAMs through transcriptionally inferred metabolic programs and predicted ligand–receptor communication in colorectal cancer, with in situ support from spatial transcriptomics. We further derive and externally validate a prognostic model based on this axis for risk stratification. Together, these findings support a putative pro-tumoral metabolic association in the tumor microenvironment and nominate testable targets for future functional validation.

## Data Availability

The datasets presented in this study can be found in online repositories. The names of the repositories can be found in the article. The code is available at https://github.com/xueyu2025/CRC-scRNAseq-analysis (accessed on 12 May 2026).

## Supplementary Materials

The following supporting information can be downloaded at: https://www.mdpi.com/article/10.3390/cancers18111755/s1, Figure S1. Single-cell profiling and survival analysis of colorectal cancer infiltration; Figure S2. Pathway enrichment and transcription factor analysis in myeloid subtypes; Figure S3. Metabolic activity and pathway analysis in myeloid subtypes; Figure S4. Differential expression signatures across 41 TCGA-COAD tumor and paired normal samples; Figure S5. Metabolic pathway activities across tumor epithelial and myeloid subtypes with spatial and molecular validation; Figure S6. Ligand-receptor interactions and regulatory potential between *SLC6A20*+ epithelial cells and *SPP1*+ TAMs; Figure S7. Prognostic evaluation and validation of gene-based prediction models for risk stratification and survival prediction; Table S1. Public datasets and accession numbers included in this study; Table S2. Differential genes between tumor and normal samples in TCGA data.
