# Supplementary material for: Integrated Single-Cell and Spatial Transcriptomic Analysis Identifies Putative Metabolic Crosstalk Between SPP1+ TAMs and SLC6A20+ Epithelial Cells in Colorectal Cancer
Source: Cancers (Basel). 2026 May 27;18(11):1755. doi: 10.3390/cancers18111755 (PMC13255607; doi:10.3390/cancers18111755)
Supplement: Supplementary file 1 [file cancers-18-01755-s001.zip › Supplementary Figures.pdf]

Supplementary Figure Legends

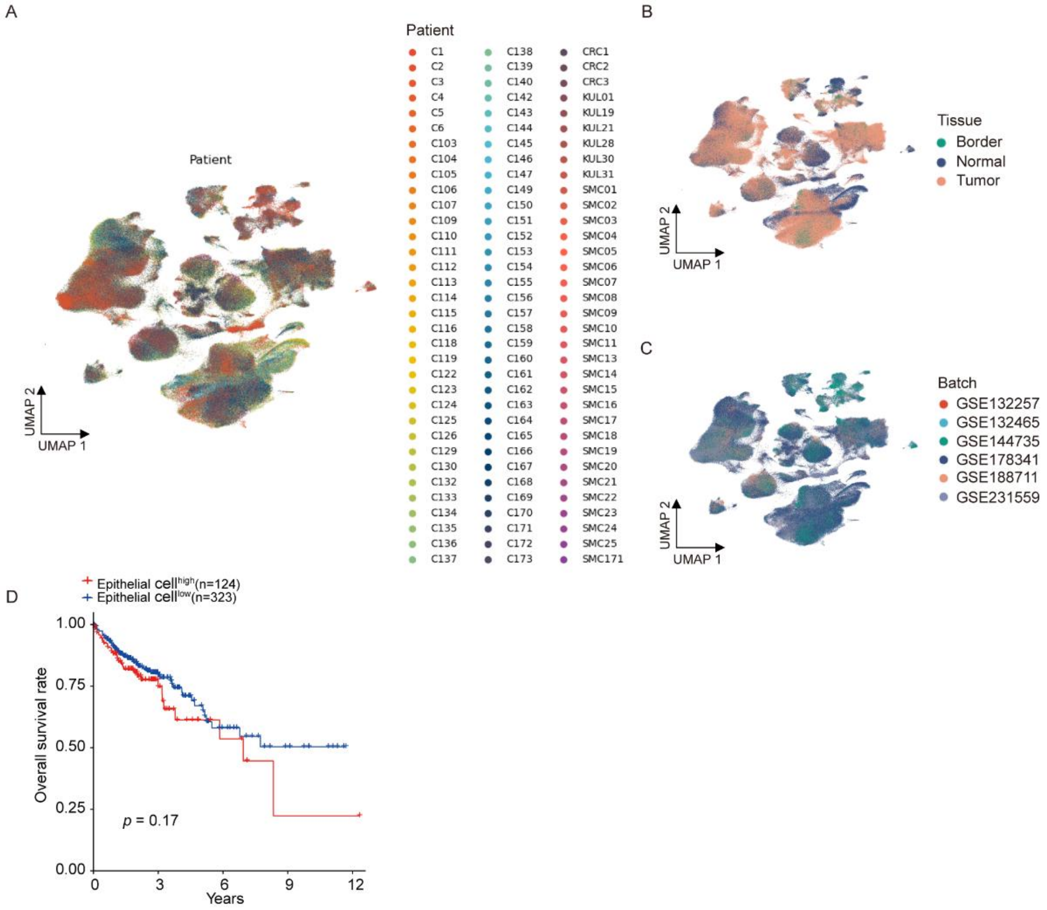

Supplementary Figure S1. Single-cell profiling and survival analysis of colorectal cancer infiltration.

(**A**) UMAP plot of cells profiled by scRNA-seq colored by patient. (**B**) UMAP plot of cells profiled by scRNA-seq colored by tissue. (**C**) UMAP plot of cells profiled by scRNA-seq colored by dataset. (**D**) Kaplan–Meier plots showing no significant difference in survival between patients with high and low infiltration of epithelial cells.

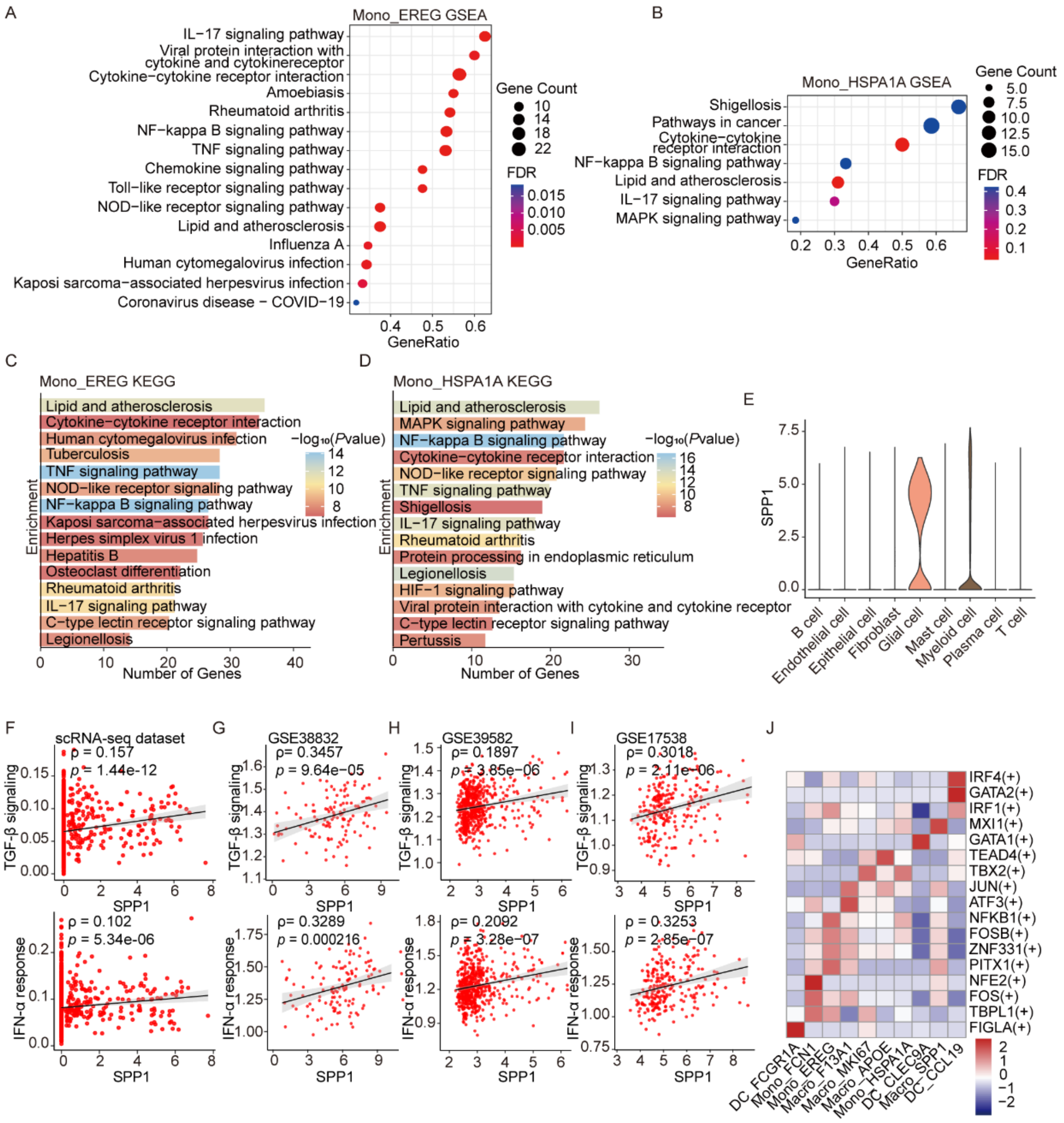

Supplementary Figure S2. Pathway enrichment and transcription factor analysis in myeloid subtypes.

(**A**) GSEA analysis of upregulated pathways enriched in Mono_EREG. (**B**) GSEA analysis of upregulated pathways enriched in Mono_HSPA1A. (**C**) KEGG enrichment analysis of the top 15 upregulated pathways in Mono_EREG. (**D**) KEGG enrichment analysis of the top 15 upregulated pathways in Mono_HSPA1A. (**E**) Violin plots showing *SPP1* expression across nine major cell types. (F-I) Spearman correlation between *SPP1* expression and ssGSEA scores for TGF-β signaling and IFN-α response in the integrated scRNA-seq dataset (**F**), GSE38832 (**G**), GSE39582 (**H**) and GSE17538 (**I**). (**J**) Heatmap of transcription factor regulon specificity (RSS) across myeloid subtypes.

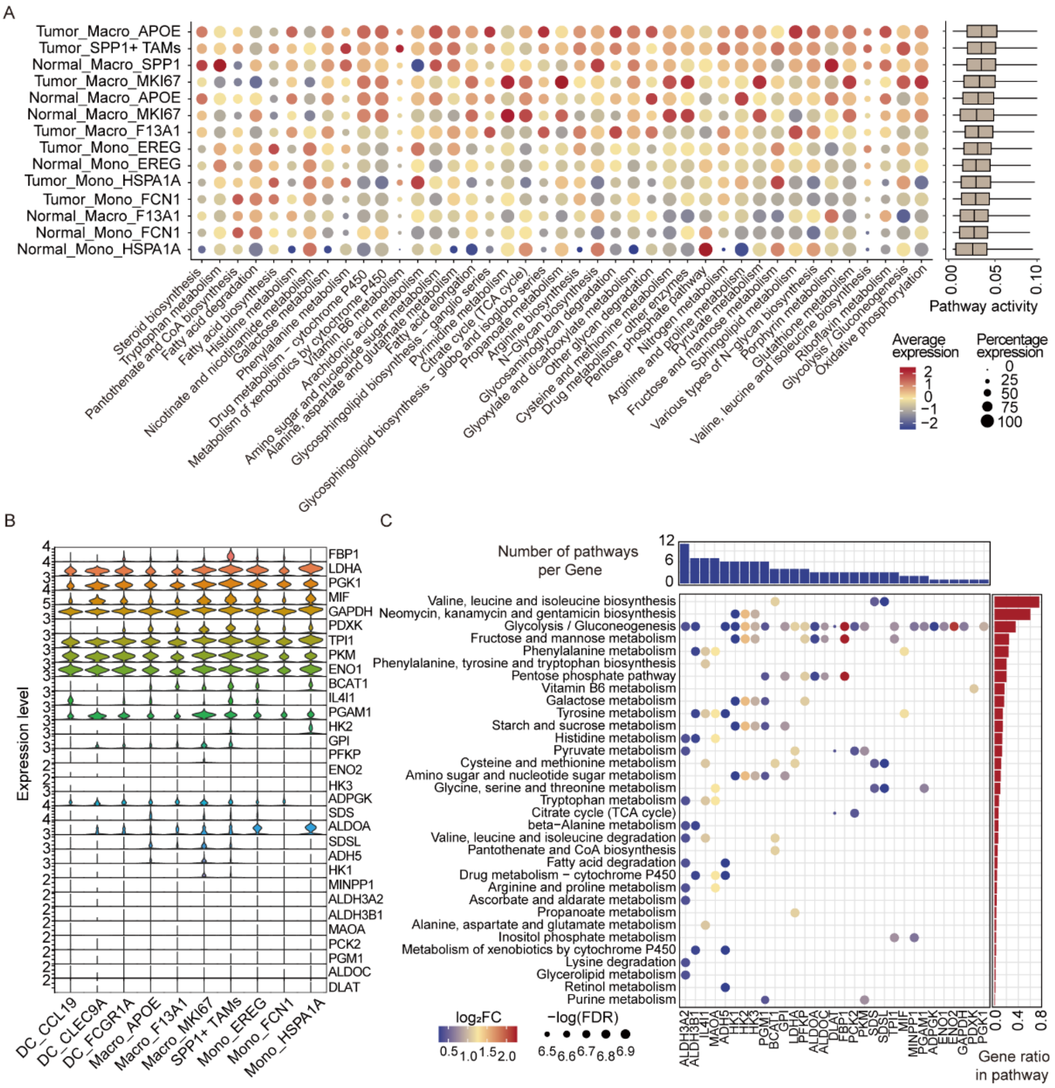

Supplementary Figure S3. Metabolic activity and pathway analysis in myeloid subtypes.

(**A**) The metabolic activity analysis of myeloid cells shows that Tumor_Macro_APOE and Tumor_*SPP1*+ TAMs (Tumor-derived *SPP1*+ macrophages) have the highest metabolic scores. (**B**) Expression of 31 metabolism-related DEGs in the *SPP1*+ TAMs subset across myeloid subtypes. (**C**) Distribution of 31 differentially expressed genes (DEGs) from the *SPP1*+ TAMs across 82 KEGG metabolic pathways. The top bar chart depicts the number of enriched pathways per DEG, ordered by descending frequency. The central dot plot maps each DEG (x-axis) to KEGG metabolic pathways (y-axis). The right bar chart represents the DEG-to-total gene ratio for each pathway.

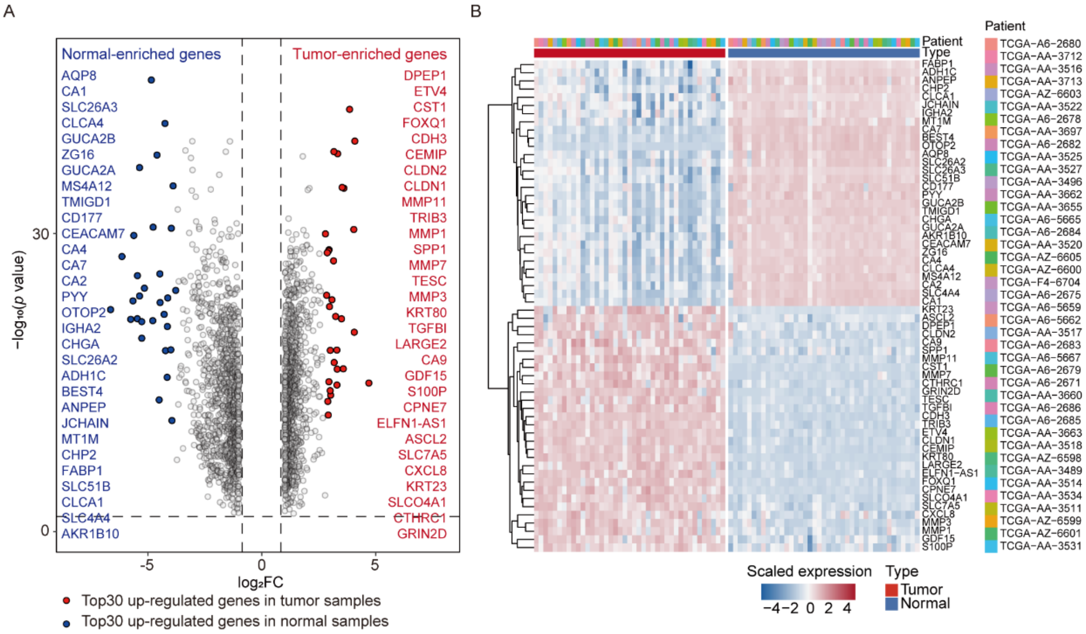

Supplementary Figure S4. Differential expression signatures across 41 TCGA-COAD tumor and paired normal samples.

(**A**) Volcano plot of 41 paired TCGA-COAD tumors and adjacent normal tissues highlighting the top 30 tumor-enriched genes and top 30 normal-enriched genes, where genes were considered tumor-enriched when log2FC > 1 with FDR-adjusted $p < 0.05$ and normal-enriched when log2FC < −1 with FDR-adjusted $p < 0.05$, and dashed lines denote |log2FC| = 1 and $p = 0.05$ (displayed as $-\log_{10}(p \text{ value})$). (**B**) Heatmap of the top 30 genes upregulated in tumor and the top 30 genes upregulated in normal across 41 paired TCGA-COAD samples (‘01A’: tumor; ‘11A’: normal).

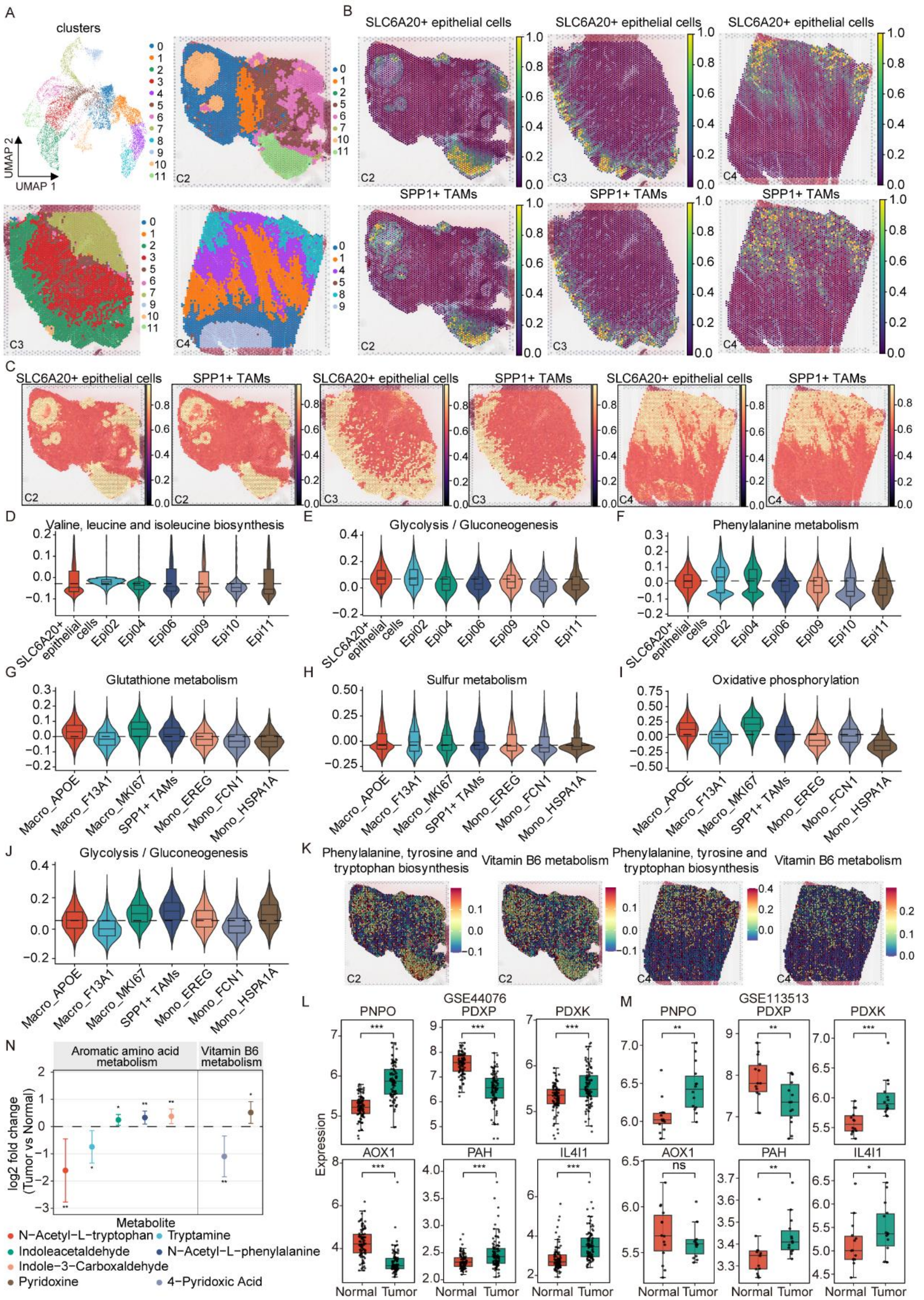

Supplementary Figure S5. Metabolic pathway activities across tumor epithelial and myeloid subtypes with spatial and molecular validation.

(**A**) UMAP of the ST data colored by clusters, with corresponding spatial cluster distribution maps for each section (C2-C4) after clustering. (**B**) Tangram inferred spatial distributions of *SLC6A20*+ epithelial cells and *SPP1*+ TAMs in tissue sections C2-C4. (**C**) Cell2location inferred spatial distributions of *SLC6A20*+ epithelial cells and *SPP1*+ TAMs in tissue sections C2-C4 (D-F) Violin plot of valine, leucine

and isoleucine biosynthesis (**D**), glycolysis/gluconeogenesis (**E**) and phenylalanine metabolism (**F**) module scores across tumor epithelial subtypes. (G-J) Violin plot of glutathione metabolism (**G**), sulfur metabolism (H), oxidative phosphorylation (**I**) and phenylalanine tyrosine tryptophan biosynthesis (**J**) module scores across macrophages and monocytes subtypes. (**K**) Spatial feature plots showing pathway activity scores of vitamin B6 metabolism and phenylalanine tyrosine tryptophan biosynthesis in C2 and C4. (L–M) Boxplots showing the expression of *PNPO, PDXP, PDXK, AOX1, PAH*, and *IL4I1* in normal and tumor tissues from GSE44076 (**L**) and GSE113513 (**M**). Boxes indicate the interquartile range, center lines indicate the median, whiskers indicate 1.5 × IQR, and dots represent individual samples. Statistical significance was assessed using the Wilcoxon rank-sum test. *, $p < 0.05$; **, $p < 0.01$; ***, $p < 0.001$. (**N**) Differential metabolites associated with aromatic amino acid metabolism (phenylalanine tyrosine tryptophan biosynthesis) and vitamin B6 metabolism in tumor versus normal samples. Points indicate log2 fold change (Tumor vs Normal), with positive values representing higher abundance in tumors and negative values representing higher abundance in controls. Error bars represent approximate 95% confidence intervals. *, $p < 0.05$; **, $p < 0.01$; ***, $p < 0.001$.

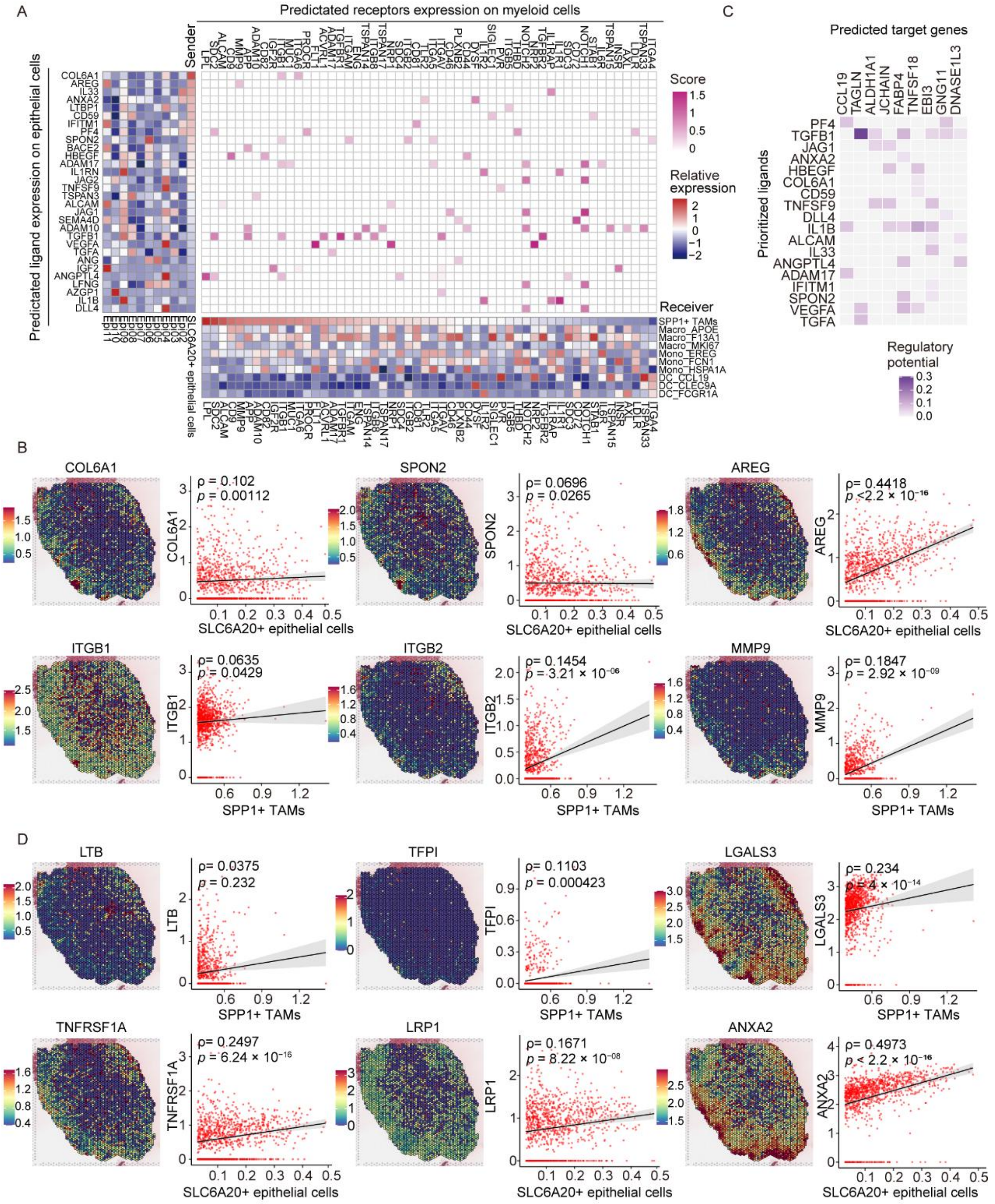

Supplementary Figure S6. Ligand-receptor interactions and regulatory potential between *SLC6A20*+ epithelial cells and *SPP1*+ TAMs.

(**A**) Heatmaps of the top predicted ligands identified by NicheNet for *SLC6A20*+ epithelial cells that may regulate *SPP1*+ TAMs (left), the relative expression of corresponding receptors across myeloid subtypes (bottom), and significant ligand−receptor pairs between *SLC6A20*+ epithelial cells and *SPP1*+ TAMs (middle). (**B**) Spatial distributions of ligand-receptor pairs COL6A1-ITGB1, SPON2-ITGB2, and AREG-MMP9 in C3. Spearman correlations between *SLC6A20*+ epithelial cells signature score and the expression of its ligands COL6A1, SPON2, and AREG, as well as between *SPP1*+ TAMs signature score and the expression of its receptors ITGB1, ITGB2, and MMP9. (**C**) Heatmap showing regulatory potential

of top ligands and the downstream target genes in *SPP1*+ TAMs. (**D**) Spatial distributions of ligand-receptor pairs LTB-TNFRSF1A, TFPI-LRP1, and LGALS3-ANXA2 in C3. Spearman correlations between *SPP1*+ TAMs signature score and the expression of its ligands LTB, TFPI, and LGALS3, as well as between *SLC6A20*+ epithelial cells signature score and the expression of its receptors TNFRSF1A, LRP1, and ANXA2.

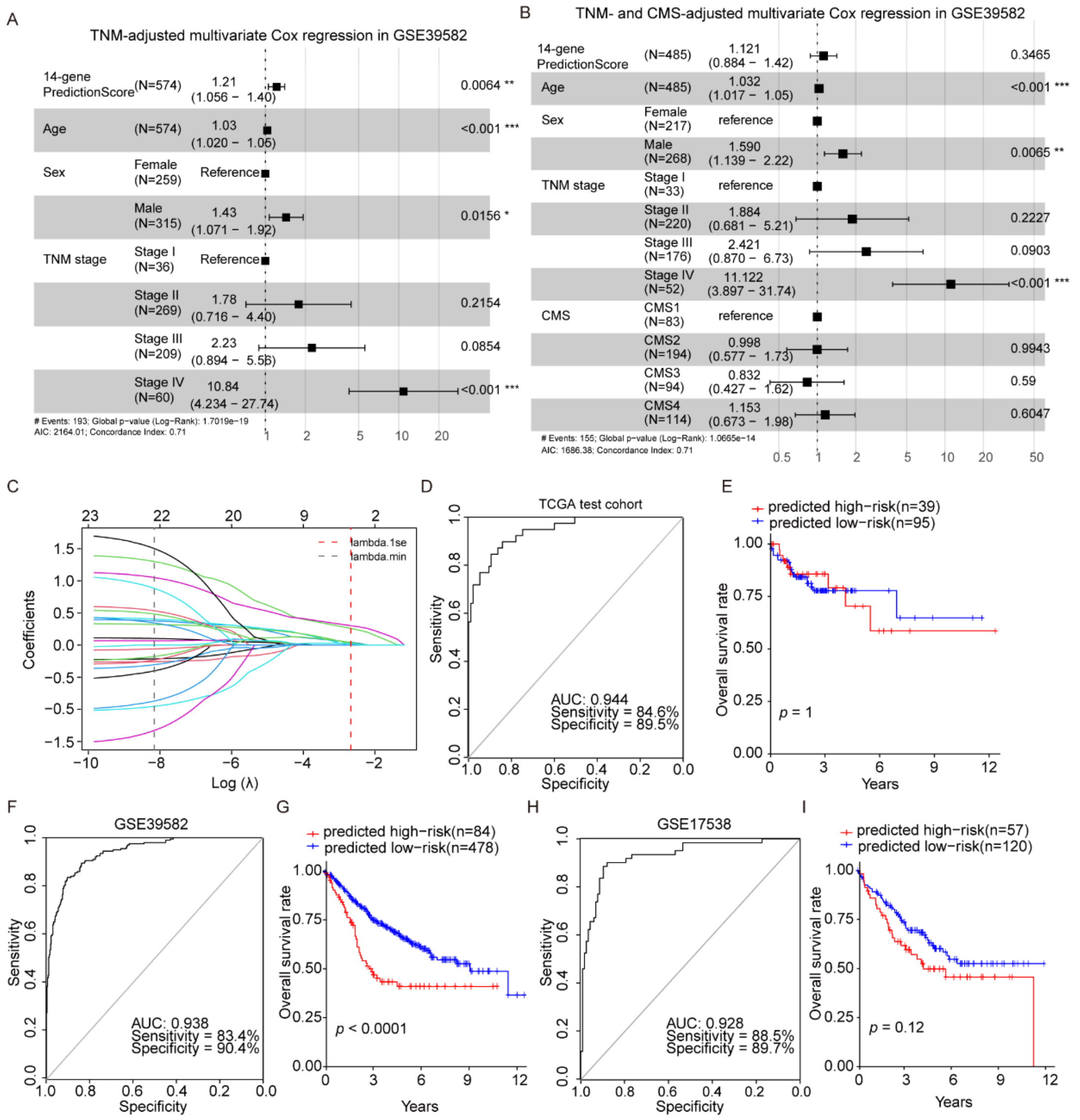

Supplementary Figure S7. Prognostic evaluation and validation of gene-based prediction models for risk stratification and survival prediction.

(**A**) Forest plot showing HRs and 95% CIs for the standardized 14-gene PredictionScore, age, sex, and TNM stage; female sex and Stage I were used as references. (**B**) Forest plot showing HRs and 95% CIs for the standardized 14-gene PredictionScore, age, sex, TNM stage, and CMS subtype; female sex, Stage I, and CMS1 were used as references. (**C**) LASSO coefficient profiles of the candidate genes. The six-gene LASSO prediction score was calculated as: Prediction-Score = 0.260 × *SFRP2* + 0.042 × *SPP1* + 0.302 × *MARCO* + 0.021 × *SERPINB2* + 0.038 × *COMP* + 0.020 × *TNC* − 6.33. (**D**) Receiver operating characteristic (ROC) curves for predictions based on the expression levels of 6 genes in the TCGA test cohort (n = 134). (**E**) Kaplan–Meier curve (log-rank test) showing overall survival (OS) for the predicted

high-risk and low-risk groups in the TCGA test cohort. (**F**, **H**) ROC curves for predictions based on the expression levels of 6 genes in the validation cohorts GSE39582 (**F**) (n = 562) and GSE17538 (H) (n = 177). (**G**, **I**) Kaplan–Meier curves (log-rank test) showing OS for the predicted high-risk and low-risk groups in the validation cohorts GSE39582 (**G**) and GSE17538 (I).
